# TMT proteomics analysis of a pseudocereal crop, quinoa (*Chenopodium quinoa* Willd.), during seed maturation

**DOI:** 10.3389/fpls.2022.975073

**Published:** 2022-11-08

**Authors:** Zhi-Jun Shen, Su-Xia Xu, Qing-Yun Huang, Zi-Yang Li, Yi-Ding Xu, Chun-Song Lin, Yi-Jin Huang

**Affiliations:** ^1^ Fujian Key Laboratory of Subtropical Plant Physiology and Biochemistry, Fujian Institute of Subtropical Botany, Xiamen, China; ^2^ Institute of Gene Science for Bamboo and Rattan Resources, International Center for Bamboo and Rattan, Beijing, China; ^3^ Landscape Architecture and Landscape Research Branch, China Academy of Urban Planning and Design, Beijing, China; ^4^ Department of Dermatology, The First Affiliated Hospital of Xiamen University, Xiamen, China

**Keywords:** quinoa, seed maturation, TMT proteomics, regulation mechanism, PRM

## Abstract

Quinoa (*Chenopodium quinoa* Willd.), an Andean native crop, is increasingly popular around the world due to its high nutritional content and stress tolerance. The production and the popularity of this strategic global food are greatly restricted by many limiting factors, such as seed pre-harvest sprouting, bitter saponin, *etc*. To solve these problems, the underlying mechanism of seed maturation in quinoa needs to be investigated. In this study, based on the investigation of morphological characteristics, a quantitative analysis of its global proteome was conducted using the combinational proteomics of tandem mass tag (TMT) labeling and parallel reaction monitoring (PRM). The proteome changes related to quinoa seed maturation conversion were monitored to aid its genetic improvement. Typical changes of morphological characteristics were discovered during seed maturation, including mean grain diameter, mean grain thickness, mean hundred-grain weight, palea, episperm color, *etc*. With TMT proteomics analysis, 581 differentially accumulated proteins (DAPs) were identified. Functional classification analysis and Gene Ontology enrichment analysis showed that most DAPs involved in photosynthesis were downregulated, indicating low levels of photosynthesis. DAPs that participated in glycolysis, such as glyceraldehyde-3-phosphate dehydrogenase, pyruvate decarboxylase, and alcohol dehydrogenase, were upregulated to fulfill the increasing requirement of energy consumption during maturation conversion. The storage proteins, such as globulins, legumins, vicilins, and oleosin, were also increased significantly during maturation conversion. Protein–protein interaction analysis and function annotation revealed that the upregulation of oleosin, oil body-associated proteins, and acyl-coenzyme A oxidase 2 resulted in the accumulation of oil in quinoa seeds. The downregulation of β-amyrin 28-oxidase was observed, indicating the decreasing saponin content, during maturation, which makes the quinoa “sweet”. By the PRM and qRT-PCR analysis, the expression patterns of most selected DAPs were consistent with the result of TMT proteomics. Our study enhanced the understanding of the maturation conversion in quinoa. This might be the first and most important step toward the genetic improvement of quinoa.

## Introduction

Quinoa (*Chenopodium quinoa* Willd.) is a tetraploid (2n = 4x = 36) native crop that has been used as food and medicine by the indigenous people of the Andes for 8,000 years (Schlick and Bubenheim, 1996; Bhargava et al., 2006). Compared to common cereal grains, the quinoa seed contains a considerably higher protein content and an excellent balance of essential amino acids, lipids, carbohydrates, dietary fibers, vitamins, and minerals; their nutritional quality is similar to that of milk (Wu et al., 2014; Beloshapka et al., 2016). Besides this, quinoa is gluten-free and has a low glycemic index; thus, it is suitable for people with celiac disease (Zevallos et al., 2014). Quinoa also has high salt tolerance and can grow under extremely dry conditions (Ruiz et al., 2016; Walter et al., 2016). The specific adaptations of this species to certain geographical areas gave rise to different ecotypes. Quinoa has adapted to areas with high rainfall (precipitation of 2,000 mm/year) and areas with extreme drought stress (precipitation of 150 mm/year) (Fuentes et al., 2012). It can be used as a drought-tolerant, salt-tolerant, and nutritious staple food. However, the quinoa supply is highly insufficient, especially considering that many people are suffering from starvation or malnutrition all over the world (FAO et al., 2014). Despite its agronomic potential, quinoa is an underutilized crop. It might be used to enhance the global food security for a growing world population (Massawe et al., 2016). After the international year of quinoa in 2013, quinoa has gained increasing attention around the world regarding its profuse nutrition and ability to withstand extreme conditions (Bazile et al., 2016). Today, quinoa is grown in more than 120 countries around the world, which will provide a solid guarantee for world food security (Alandia et al., 2020).

There is a common concern about nutrition accumulation in quinoa production. As a nutritious food, quinoa is characterized by seed storage proteins, carbohydrates, starch, saponins, vitamins, and minerals (Wu et al., 2014). Among them, seed storage proteins are mainly responsible for seed germination (Aloisi et al., 2016), and they also make quinoa nutritious. However, quinoa still has some unpleasant traits, such as pre-harvest sprouting and bitterness of the seed coat (Lopes et al., 2019). Pre-harvest sprouting is one of the major problems in quinoa production which irritates farmers (Lintschinger et al., 1997; Lopes et al., 2019). The identification of pre-harvest sprouting-related proteins might facilitate genetic improvement during seed maturation (Burrieza et al., 2019). Besides this, quinoa seeds contain a mixture of triterpene glycosides called “saponins” (David et al., 2017). Saponins are another major problem in quinoa production. Although saponins are beneficial for plant growth (Kuljanabhagavad and Wink, 2009), they impart bitterness to the grain (Fiallos-Jurado et al., 2016). Therefore, saponins must be removed before human consumption. An investigation on saponin accumulation during seed maturation might help to modify the grain flavor in future quinoa production.

Thus, to promote the genetic improvement of quinoa, identifying these typical proteins that are required during seed maturation is necessary. Proteomics is an efficient tool that can help to identify reliable protein markers (David et al., 2017). A high-quality genome sequence for quinoa was published. Building on that, here we first conducted the combinational proteomics of TMT and parallel reaction monitoring (PRM) (Peterson et al., 2012). Based on these approaches, we assessed the changes in the proteomics related to seed maturation conversion in *Chenopodium quinoa* Willd. for enhancing the genetic improvement in quinoa.

## Materials and methods

### Plant materials and morphological observation

The experiments were conducted at Fujian Institute of Subtropical Botany’s Quinoa Garden, located in Xiamen, China (118°04′04″ E, 24°26′46″ N, annual average temperature of 22°C), at an elevation of 63 m above sea level. The site receives an average rainfall of 1,200 mm/year.

The biosample accession code PI596293 was obtained from Plant Germplasm Quarantine Center, USDA. PI596293, which named as COLORADO 407D, is a native ecotype from Colorado, USA, and is early maturing. COLORADO 407D was characterized by a central axis with secondary and tertiary axes. Its amaranthiform is large, and the inflorescences are compact and glamorous. The seeds of COLORADO 407D are large and sweet, and they are yellow. We sowed the seeds on October 26, 2017, eared them after December 10, and harvested them on January 26, 2018. The inflorescence was collected in both developing stages. The first stage (stage I) was set in 72 days after sowing, and the second stage (stage II) was set in 84 days after sowing. The grains from stage I were immature seeds (IS) on the eve of seed maturation conversion. The grains from stage II were mature seeds (MS), which were edible and dry. At least 15 quinoa plants were randomly selected, and their morphological changes from stage I to stage II were continuously recorded, such as the mean plant height, mean diameter of inflorescence, mean height of inflorescence, leaf color, leaf axil color, stem streak color, palea color, and episperm color. For the mean grain diameter, mean grain thickness, and mean hundred-grain weight, the inflorescences were cut from at least 15 quinoa plants, and the seeds were collected to measure the characteristics. Each assay was tested triplicate (at least 45 quinoa plants for each stage).

### Protein extraction

Inflorescences from uniform and healthy quinoa plants were randomly selected for protein extraction. The whole inflorescences from different developmental stages were collected, quickly frozen in liquid nitrogen, and stored at –80°C. The sample was ground into powder using liquid nitrogen and transferred to a 5-ml centrifuge tube. Then, fourfold volumes of lysis buffer (8 M urea, 1% Triton-100, 10 mM dithiothreitol, and 1% protease inhibitor cocktail) were added to the tube, followed by sonication three times on ice using a high-intensity ultrasonic processor (Scientz). The remaining debris was removed by centrifugation at 20,000*g* and 4°C for 10 min. Finally, the protein was precipitated with cold 20% trichloroacetic acid for 2 h at –20°C. After centrifugation at 12,000*g* and 4°C for 10 min, the supernatant was discarded. The remaining precipitate was washed with cold acetone three times. The protein was redissolved in 8 M urea, and the protein concentration was determined using a BCA kit (Solarbio Technology Co. Ltd., Beijing, China) following the manufacturer’s instructions. Three independent biological replicates for each stage were analyzed.

### Trypsin digestion

The protein solution was reduced with 5 mM dithiothreitol for 30 min at 56°C and alkylated with 11 mM iodoacetamide for 15 min at room temperature in darkness. The protein sample was diluted to the urea concentration of less than 2 M. Finally, trypsin was added at a 1:50 trypsin-to-protein mass ratio for the first digestion overnight at 37°C and a 1:100 trypsin-to-protein mass ratio for another digestion for 4 h.

### TMT labeling

The peptide was desalted using a Strata X C18 SPE column (Phenomenex) and vacuum-dried after trypsin digestion. Following the manufacturer’s protocol of the TMT kit, the peptide was reconstituted and processed in 0.5 M TEAB solution. Briefly, the peptide was thawed and reconstituted in acetonitrile, incubated with one unit of TMT reagent for 2 h at room temperature, then pooled, desalted, and dried by vacuum centrifugation.

### HPLC fractionation

The tryptic peptides were digested into fractions by high-pH reversed-phase HPLC using an Agilent 300Extend C18 column (5-µm particles, 4.6-mm inner diameter (i.d.), and 250-mm length). Then, the peptides were first separated with a gradient of 8 to 32% acetonitrile (pH 9.0) over 60 min into 60 fractions. Finally, the peptides were divided into 18 fractions and vacuum-dried.

### LC–MS/MS analysis

The peptides were dissolved in 0.1% formic acid (solvent A) before the tryptic peptides were loaded onto a homemade reversed-phase analytical column (15-cm length, 75 µm i.d.). The gradient consisted of an increase from 6 to 23% solvent B (0.1% formic acid in 98% acetonitrile) over 26 min, 23 to 35% in 8 min, and increased to 80% in 3 min, followed by holding at 80% for the last 3 min, at a constant flow rate of 400 nl/min on an EASY-nLC 1000 UPLC system. The peptides were subjected to the NSI source followed by tandem mass spectrometry (MS/MS) in Q Exactive™ Plus (Thermo) coupled online to the UPLC. The electrospray voltage applied was 2.0 kV. The m/z scan range was 350 to 1,800 for the full scan, and intact peptides were detected in the Orbitrap at a resolution of 70,000. The peptides were then selected for MS/MS using the NCE setting as 28, and the fragments were detected in the Orbitrap at a resolution of 17,500. A data-independent procedure was used, which alternated between one MS scan followed by 20 MS/MS scans with 15.0-s dynamic exclusion. Automatic gain control (AGC) was set at 5E4 for full MS and 1E5 for MS/MS. The fixed first mass was set as 100 m/z. The information on basic mass spectrometry is provided in **Supplementary Table S2**.

### Database search

The obtained MS/MS data were processed using the Maxquant search engine (v.1.5.2.8). Tandem mass spectra were searched against the quinoa genome database (biosample accession code SAMN04338310, submitted on December 15, 2015, https://www.ncbi.nlm.nih.gov/biosample/?term=SAMN04338310) concatenated with the reverse decoy database. The mass tolerance for precursor ions was set as 20 ppm for the first search and 5 ppm for the main search. The mass tolerance for fragment ions was set as 0.02 Da. Trypsin/P was specified as a cleavage enzyme allowing up to two missing cleavages. Carbamidomethyl on Cys was specified as fixed modification, and oxidation on Met was specified as variable modifications. The false discovery rate was adjusted to <1%, and the minimum score for peptides was set to >40.

### Protein function and subcellular localization analysis

The function of DAPs was searched against the UniProt, NCBI, and Target P databases. The subcellular localization of DAPs was performed using WoLF PSORT, a subcellular localization prediction software. An updated version of PSORT/PSORT II was used for the prediction of eukaryotic sequences and molecular function. A corrected *p*-value of <0.05 was considered to be significant. The results of the function annotation are presented in **Supplementary Table S3**.

### Protein–protein interaction analysis and gene ontology enrichment analysis

The protein–protein interaction (PPI) network of DAPs was constructed using STRING 11.5 (https://cn.string-db.org/). To match the proteins in the database as much as possible, the DAPs identified in our study were transformed to the corresponding proteins in *Arabidopsis thaliana*, and the interaction network was constructed (the detailed information on each DAP is provided in **Supplementary Table S4**). The interaction network was redrawn using Cytoscape v3.5.0. ClueGO was used to analyze the GO enrichment of DAPs.

### PRM validation for targeted MS analysis

To determine the reliability of the results of sequencing, the original protein samples were applied to the same liquid chromatography (LC)–mass spectrometry (MS) system used above. Based on the results from the abovementioned assessment, 17 proteins were selected for the PRM assay. The methods of protein extraction and trypsin digestion for the PRM analysis were similar to that described for the TMT proteomics. The LC–MS/MS analysis and the data analysis of PRM were performed following the methods described by Hashimoto et al. (2019) with some modifications. The tryptic peptides were dissolved in 0.1% formic acid (solvent A) and also loaded onto a reversed-phase analytical column. The gradient consisted of an increase from 6 to 23% solvent B (0.1% formic acid in 90% acetonitrile) over 38 min, 23 to 35% in 14 min, and increased to 80% in 4 min, followed by holding at 80% for the last 4 min, at a constant flow rate of 400 nl/min on an EASY-nLC 1000 UPLC system. The peptides were subjected to the NSI source, followed by tandem mass spectrometry (MS/MS) in Q Exactive™ Plus (Thermo) coupled online to the UPLC. The electrospray voltage applied was 2.0 kV. The m/z scan range was 300 to 1,000 for the full scan, and intact peptides were detected in the Orbitrap at a resolution of 35,000. The peptides were then selected for MS/MS using the NCE setting as 27, and the fragments were detected in the Orbitrap at a resolution of 17,500. A data-independent procedure alternated between one MS scan followed by 20 MS/MS scans. AGC was set at 3E6 for full MS and 1E5 for MS/MS. The maximum IT was set at 20 ms for full MS and auto for MS/MS. The isolation window for MS/MS was set at 2.0 m/z. The MS/MS data were processed using Skyline (v. 3.6). For the peptide settings, trypsin (KR/P) was set as the enzyme, and the maximum missed cleavage was two. The length of the peptide was set as 8–25. The variable modification was set as carbamidomethyl on Cys and oxidation on Met, and the maximum variable modification was set as three. For the transitional settings, precursor charges were set as 2 and 3, ion charges were set as 1 and 2, and ion types were set as b, y, and p. The product ions were set from ion 3 to the last ion, and the ion match tolerance was set as 0.02 Da. The information on basic mass spectrometry is provided in **Supplementary Table S5**.

### RNA extraction and qRT-PCR

Total RNA was extracted from quinoa inflorescence (0.2 g) in different developmental stages with 1 ml of Trizol reagent (TaKaRa, Dalian, China) following the manufacturer’s protocol. The Moloney murine leukemia *virus* reverse transcriptase from TaKaRa was used for the synthesis of first-strand cDNA. The expression levels of the selected genes were detected using the gene-specific primers (listed in **Supplementary Table S6**) on Bio-Rad CFX96 Touch (Bio-Rad, USA). The 2^–△△Ct^ method was used to calculate the relative quantification values of each gene. Tub-6 was used as the housekeeping gene.

### Statistical analysis

For analyzing the data on morphological observation and qRT-PCR, at least three replicates were used. For the proteomics analysis, three independent biological replicates were included. The correlation analyses of the 17 selected DAPs based on the results of TMT and PRM were performed by using SPSS 19.0 (SPSS, Chicago, IL, USA). The statistical significance was tested by performing a one-way ANOVA or Duncan’s post-test (*P* < 0.05) for multiple comparisons using SPSS 19.0 (SPSS, Chicago, IL, USA). The data presented in the figures and tables are expressed as mean ± SE.

## Results

### Morphological comparison between two developing stages

**Figure 1:**
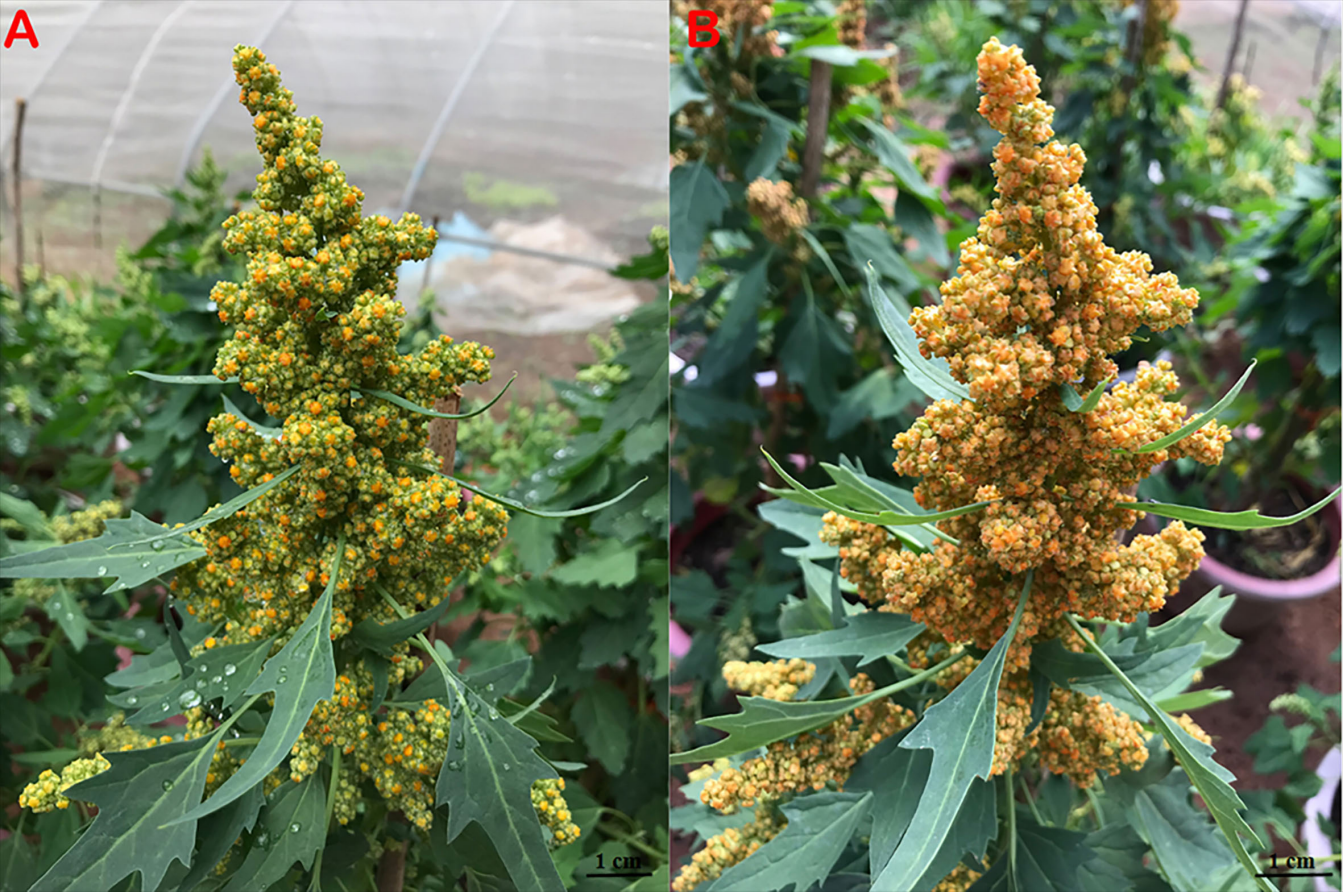
Images of quinoa seeds in different stages. **(A)** Immature seeds of quinoa in stage I and **(B)** mature seeds of quinoa in stage II.

The quinoa seeds in stage II were fuller than the seeds in stage I (**Figure 1**). The morphological characteristics of quinoa in different developmental stages showed that the mean grain diameter, mean grain thickness, and mean hundred-grain weight were significantly higher in stage II than in stage I. The palea color in stage I was green and subsequently turned yellow in stage II (**Table 1**). The episperm color changed from yellow to orange during seed maturation (**Figure 1** and **Table 1**). Based on these results, the seeds of quinoa in stage I and stage II were considered to be immature seeds (IS) and mature seeds (MS), respectively.

**Table 1:** Morphological characteristics of the plant in two development stages.

Stage	Stage I	Stage II
Mean plant height (cm)	111.2 ± 1.46	107.1 ± 1.22
Mean diameter of inflorescence (cm)	5.4 ± 0.62	5.6 ± 0.53
Mean height of inflorescence (cm)	30.5 ± 0.73	32 ± 1.37
Leaf color	Green	Green
Leaf axil color	Green	Green
Stem streaks color	Green	Green
Mean grain diameter (mm)	2.2 ± 0.07	2.5 ± 0.11
Mean grain thickness (mm)	1.1 ± 0.05	1.4 ± 0.14
Mean hundred-grain weight (g)	0.22 ± 0.05	0.38 ± 0.08
Palea color	Green	Yellow
Episperm color	Yellow	Orange
Days after sewing	72	84

### Identification of differentially accumulated proteins

**Figure 2:**
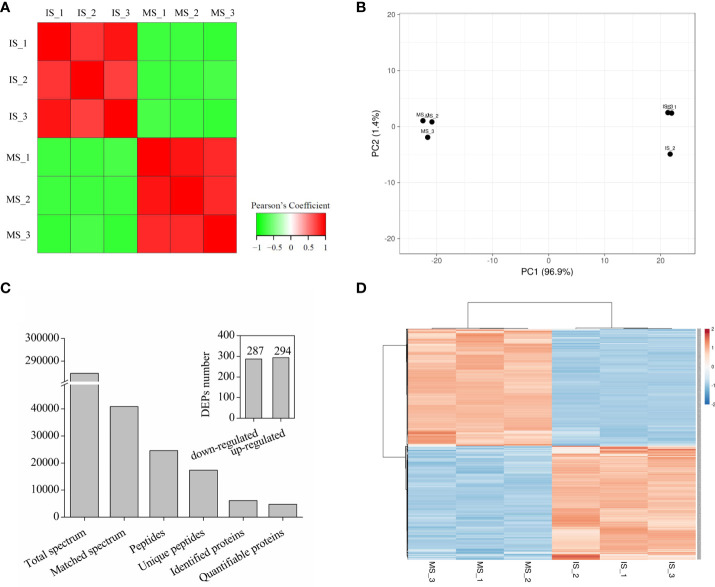
Summary of tandem mass tag proteomics analysis in quinoa seed from different developmental stages. **(A)** Pearson’s correlation coefficient was calculated from three biological replicates for each biotype/treatment. **(B)** Principal component analysis of quinoa seed in different stages. **(C)** Summary of the MS/MS spectrum and identified differentially accumulated proteins (DAPs). **(D)** Hierarchical clustering analysis of all DAPs in quinoa seed from different developmental stages.

**Figure 3:**
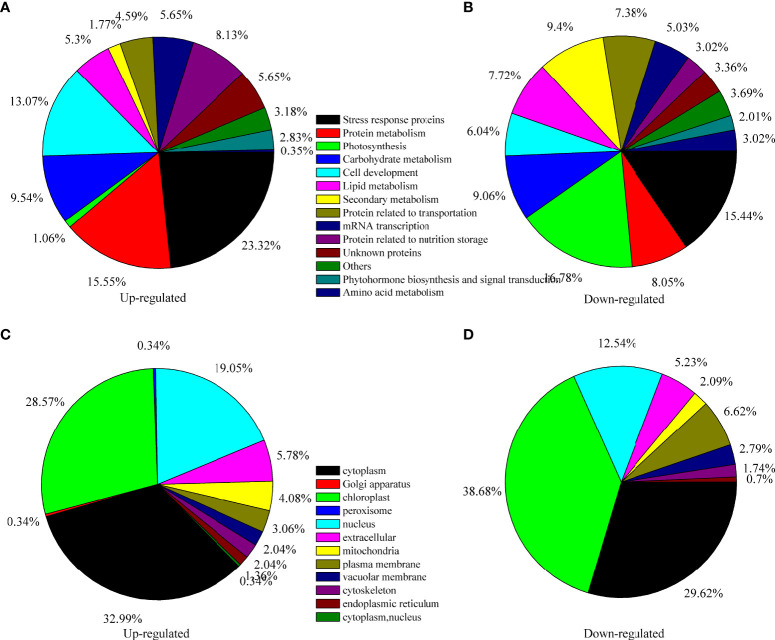
Functional classification and subcellular localization of all differentially accumulated proteins (DAPs) in quinoa seed from different developmental stages. **(A)** Function classification of upregulated DAPs, **(B)** function classification of downregulated DAPs, **(C)** subcellular localization of upregulated DAPs, and **(D)** subcellular localization of downregulated DAPs.

To further investigate the underlying mechanism of seed maturation, TMT proteomics analysis was performed. With Pearson’s correlation analysis, a positive correlation was observed among the same samples, and a negative correlation was observed between the immature seeds (IS) and mature seeds (MS) (**Figure 2A**), indicating a fine replication in the IS or MS but a significant difference between IS and MS (**Figure 2B**). Based on the HPLC and LC–MS/MS analysis, 284,587 spectra were obtained, and 40,889 spectra were identified in the database (**Figure 2C**). Among these matched spectra, 24,587 peptides and 17,362 unique peptides were obtained, respectively (**Figure 2C**). After annotation, 6,097 proteins were identified, and 4,770 proteins could be used for quantitative analysis (**Figure 2C**). The ratios of each protein in the IS and MS were calculated, and the proteins with more than twofold change (*p* < 0.05) were identified as differentially accumulated proteins (DAPs). A total of 581 proteins were identified as DAPs. Among them, 287 DAPs were downregulated and 294 DAPs were upregulated (**Figure 2C**). The expression profile of DAPs is presented in **Figure 2D** based on hierarchical clustering analysis (**Figure 2D**). The detailed information on each DAP is presented in **Supplementary Table S1**. According to biological function analysis, all DAPs were divided into 14 functional categories (**Figures 3A, B**). Most DAPs involved in stress response proteins, protein metabolism, cell development, and protein related to nutrition storage were upregulated. On the contrary, the DAPs involved in photosynthesis, lipid metabolism, and secondary metabolism and the proteins related to transportation and amino acid metabolism were mostly downregulated (**Figures 3A, B**). For the subcellular localization of DAPs, most of them were mainly located in the cytoplasm, chloroplast, and nucleus (**Figures 3C, D**). The DAPs located in the chloroplast were mostly downregulated.

The DAPs involved in cell development and the proteins related to nutrition storage are presented in **Table 2**. Most DAPs were significantly upregulated in the two functional categories, including cell development and proteins related to nutrition storage (**Table 2**). For example, 23 of 32 DAPs were upregulated for the proteins related to nutrition storage, such as oleosins, globulin seed storage proteins, vignains, vicilins, and legumins. Out of 55 DAPs, 37 DAPs were upregulated in cell development (**Table 2**). Most of the upregulated proteins were closely related to seed maturation, such as seed biotin-containing protein SBP65 (SBP65) and late embryogenesis abundant proteins (LEA).

**Table 2:** Differentially accumulated proteins (DAPs) participating in cell development and protein related to nutrition storage in *Chenopodium quinoa* Willd.

Accession number	Protein description	MW (kDa)	Score	Coverage (%)	Peptides	Regulated type	*P*-value
Protein related to nutrition storage							
AUR62019367-RA	bark storage protein A	35.87	19.85	15.9	5	Down	0.000016
AUR62006123-RA	bark storage protein A	35.05	101.77	41.8	11	Down	0.000037
AUR62019363-RA	bark storage protein A	35.78	21.71	28.5	8	Down	0.000000
AUR62006122-RA	bark storage protein A	32.63	4.98	7.7	2	Down	0.009556
AUR62043514-RA	vacuolar-processing enzyme-like	64.61	10.39	5.9	4	Down	0.000142
AUR62036450-RA	BURP domain protein USPL1	51.93	3.92	6.8	3	Down	0.002599
AUR62017095-RA	BURP domain protein USPL1	62.31	16.40	16.7	7	Down	0.000037
AUR62043729-RA	BURP domain-containing protein BNM2A	46.35	52.24	24.6	11	Down	0.000056
AUR62017893-RA	BURP domain-containing protein BNM2A	49.52	5.48	21.0	11	Down	0.000916
AUR62036943-RA	oleosin 1	11.91	4.30	16.5	2	Up	0.000840
AUR62040213-RA	oleosin 1	18.27	10.16	28.7	5	Up	0.000076
AUR62008167-RA	oleosin 18.2 kDa	18.20	21.12	17.8	3	Up	0.000001
AUR62012221-RA	oleosin 18.2 kDa	19.03	39.98	30.4	8	Up	0.000018
AUR62024716-RA	11S globulin seed storage protein 2 isoform X2	52.44	323.31	46.2	22	Up	0.000656
AUR62002139-RA	13S globulin seed storage protein 1	109.47	323.31	47.0	36	Up	0.000000
AUR62015569-RA	13S globulin seed storage protein 1	51.99	119.18	28.8	10	Up	0.000016
AUR62020540-RA	2S seed storage protein	15.36	1.25	17.8	3	Up	0.000717
AUR62015663-RA	2S seed storage protein	15.55	3.68	17.7	3	Up	0.000041
AUR62022079-RA	vignain	40.42	1.51	20.8	8	Up	0.000935
AUR62024218-RA	vignain	39.15	18.42	37.6	11	Up	0.000001
AUR62005521-RA	vignain	39.30	81.48	39.5	12	Up	0.037943
AUR62003182-RA	vicilin-like seed storage protein At2 g18540	101.38	62.94	15.1	15	Up	0.000000
AUR62025011-RA	vicilin-like seed storage protein At2 g28490 isoform X2	52.10	38.09	36.5	15	Up	0.003096
AUR62006523-RA	vicilin-like seed storage protein At2 g28490 isoform X2	58.20	107.75	32.1	16	Up	0.000000
AUR62034727-RA	vicilin-like antimicrobial peptides 2–2	56.23	58.78	25.6	14	Up	0.008416
AUR62016063-RA	vicilin-like antimicrobial peptides 2–2	52.39	12.42	11.8	5	Up	0.000001
AUR62033661-RA	vicilin-like antimicrobial peptides 2–2	63.67	45.72	24.1	14	Up	0.000483
AUR62028591-RA	vicilin-like antimicrobial peptides 2–3	47.63	33.36	31.0	12	Up	0.000062
AUR62032318-RA	vicilin-like antimicrobial peptides 2–3	47.49	77.51	32.0	13	Up	0.000005
AUR62024712-RA	legumin A	51.26	54.83	47.9	19	Up	0.000000
AUR62011869-RA	legumin A	53.58	193.91	46.3	19	Up	0.000218
AUR62021514-RA	basic 7S globulin	47.01	25.87	24.4	9	Up	0.000000
Cell development							
AUR62011272-RA	DNA replication licensing factor MCM5	74.12	21.36	5.4	3	Down	0.000222
AUR62024658-RA	DNA replication licensing factor MCM7	78.55	7.35	4.2	3	Down	0.037502
AUR62039793-RA	structural maintenance of chromosome protein 4	129.58	4.13	2.2	3	Down	0.000044
AUR62043832-RA	protein POLLENLESS 3-LIKE 2	40.84	1.61	2.5	1	Down	0.000217
AUR62027754-RA	synaptonemal complex protein 1-like isoform X2	93.45	0.99	0.9	1	Down	0.000577
AUR62016318-RA	protein TSS	162.98	3.44	1.8	3	Down	0.000023
AUR62021255-RA	protein HOTHEAD	61.86	1.90	3.0	2	Down	0.000084
AUR62001255-RA	AT-rich interactive domain-containing protein 3-like	69.46	2.22	6.4	3	Down	0.000419
AUR62021123-RA	probable polygalacturonase	52.75	3.50	11.8	6	Down	0.001040
AUR62031421-RA	probable polygalacturonase	100.73	33.24	6.1	6	Down	0.000058
AUR62003605-RA	probable pectinesterase/pectinesterase inhibitor 51	29.54	4.05	9.0	3	Down	0.000800
AUR62007974-RA	probable xyloglucan endotransglucosylase/hydrolase protein 6	33.49	10.03	13.8	3	Down	0.000203
AUR62001254-RA	probable pectate lyase 18	44.74	3.66	7.7	2	Down	0.000002
AUR62003682-RA	probable glycosyltransferase At5 g03795	38.19	16.82	5.8	2	Down	0.000503
AUR62032292-RA	caffeoylshikimate esterase	32.69	11.53	12.2	3	Down	0.000064
AUR62026804-RA	basic blue protein	13.39	3.00	15.9	2	Down	0.000445
AUR62020703-RA	apyrase 2	50.68	4.22	3.9	2	Down	0.000001
AUR62001903-RA	filament-like plant protein 7	102.79	1.10	1.0	1	Down	0.012003
AUR62013778-RA	glycine-rich cell wall structural protein 1.8	21.69	74.78	37.0	4	Up	0.000082
AUR62026686-RA	glycine-rich cell wall structural protein 1.8	22.21	2.18	11.6	2	Up	0.000160
AUR62037914-RA	embryonic protein DC-8	52.53	175.90	54.1	31	Up	0.000002
AUR62040165-RA	embryonic protein DC-8	53.52	59.71	53.6	28	Up	0.000000
AUR62014787-RA	embryonic protein DC-8 isoform X1	55.54	96.78	21.7	13	Up	0.000000
AUR62042308-RA	seed biotin-containing protein SBP65	72.07	120.47	57.1	35	Up	0.000016
AUR62037387-RA	seed biotin-containing protein SBP65	69.61	241.63	67.1	38	Up	0.000004
AUR62035713-RA	seed maturation protein PM41	8.69	10.94	57.3	3	Up	0.000984
AUR62016335-RA	SNF1-related protein kinase regulatory subunit gamma-like PV42a	41.70	64.12	29.4	8	Up	0.000022
AUR62011516-RA	SNF1-related protein kinase regulatory subunit gamma-like PV42a	35.80	8.89	30.3	8	Up	0.000004
AUR62020884-RA	xyloglucan endotransglucosylase/hydrolase 2	31.76	13.81	15.8	3	Up	0.000635
AUR62010618-RA	thaumatin-like protein 1	23.33	20.79	13.5	2	Up	0.005658
AUR62034669-RA	spermatogenesis-associated protein 20 isoform X1	96.62	22.67	11.6	8	Up	0.000038
AUR62022695-RA	subtilisin-like protease SBT3.1	10.28	10.44	52.5	5	Up	0.000121
AUR62019772-RA	putative cell division cycle ATPase	86.89	23.91	9.5	7	Up	0.000000
AUR62018948-RA	protein EXORDIUM	33.81	7.61	14.6	3	Up	0.000255
AUR62027741-RA	probable xyloglucan endotransglucosylase/hydrolase protein 23	31.81	9.92	11.4	3	Up	0.000056
AUR62018906-RA	probable xyloglucan endotransglucosylase/hydrolase protein 23	31.92	6.36	10.9	3	Up	0.000016
AUR62029826-RA	mitochondrial fission protein ELM1	41.23	1.20	2.2	1	Up	0.004559
AUR62020995-RA	alpha-L-arabinofuranosidase 1	73.53	1.64	20.5	10	Up	0.000181
AUR62018438-RA	alpha-L-arabinofuranosidase 1	73.58	78.96	20.5	10	Up	0.000064
AUR62007217-RA	Em-like protein GEA6	9.08	19.90	48.8	5	Up	0.000020
AUR62004613-RA	late embryogenesis abundant protein family protein	50.64	34.85	24.5	10	Up	0.000002
AUR62022650-RA	late embryogenesis abundant protein family protein	53.52	79.37	30.8	15	Up	0.000016
AUR62011287-RA	late embryogenesis abundant protein	34.23	76.45	28.7	7	Up	0.000024
AUR62007271-RA	late embryogenesis abundant protein 18	14.67	33.92	35.6	4	Up	0.000001
AUR62018728-RA	late embryogenesis abundant protein 18	14.47	4.07	34.6	4	Up	0.000098
AUR62014840-RA	late embryogenesis abundant protein 1-like	30.79	17.50	37.5	10	Up	0.000002
AUR62032329-RA	late embryogenesis abundant protein 31	26.53	11.01	21.2	4	Up	0.003676
AUR62012039-RA	late embryogenesis abundant protein 46	17.94	25.55	29.2	5	Up	0.003119
AUR62032330-RA	late embryogenesis abundant protein 47	15.07	10.01	31.5	3	Up	0.001016
AUR62029965-RA	late embryogenesis abundant protein D-29 isoform X1	21.61	52.96	33.3	8	Up	0.000002
AUR62002551-RA	late embryogenesis abundant protein D-29 isoform X1	26.69	21.18	30.3	7	Up	0.000000
AUR62028605-RA	late embryogenesis abundant protein D-34	26.87	68.50	54.4	11	Up	0.000081
AUR62034707-RA	late embryogenesis abundant protein D-34-like	27.66	120.97	65.3	13	Up	0.000119
AUR62043549-RA	late embryogenesis abundant protein D-34-like	27.53	45.92	51.7	12	Up	0.000385
AUR62011567-RA	late embryogenesis abundant protein Dc3	19.92	45.52	50.3	12	Up	0.000000

**Figure 4:**
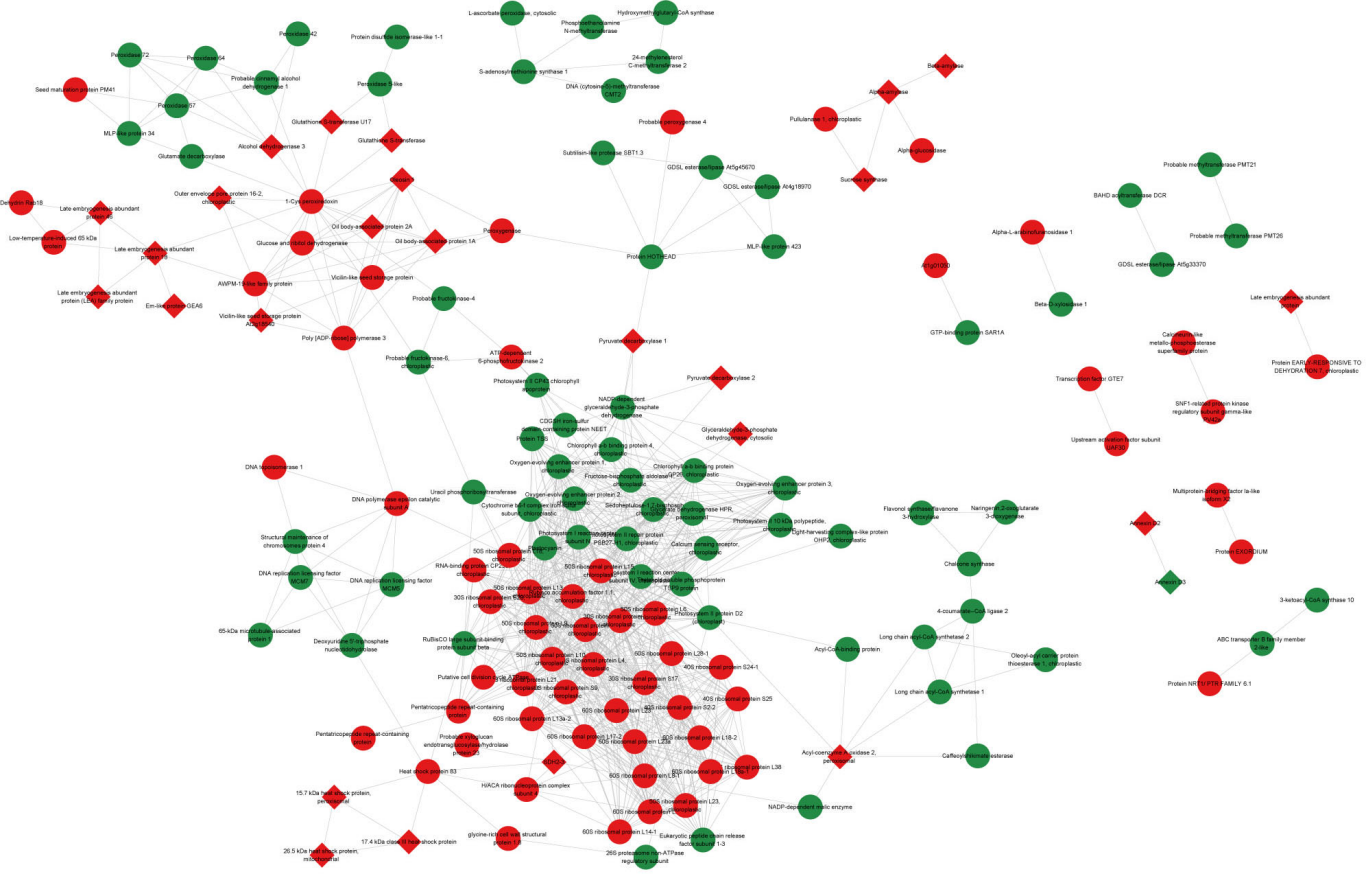
Protein–protein interaction (PPI) network of differentially accumulated proteins in quinoa seeds during maturation. The nodes in green represent downregulated proteins, and those in red represent upregulated proteins. The diamond nodes were considered to be key proteins in seed maturation by searching the relative study and database.

The interaction network of DAPs was constructed based on the PPI analysis (**Figure 4**). The 159 nodes with 895 edges were annotated in the STRING database, and 26 nodes were artificially labeled as key nodes, which were considered to play an important role in seed maturation and development. These nodes included the LEA family protein (AUR62004613-RA and AUR62022650-RA), late embryogenesis abundant protein 18 (AUR62007271-RA and AUR62018728-RA), late embryogenesis abundant protein 46 (AUR62012039-RA), late embryogenesis abundant protein (AUR62011287-RA), Em-like protein GEA6 (AUR62007217-RA), outer envelope pore protein 16–2, chloroplastic (AUR62016131-RA), alcohol dehydrogenase 3 (AUR62026788-RA), glutathione S-transferase U17 (AUR62026482-RA), glutathione S-transferase (AUR62033162-RA and AUR62008609-RA), vicilin-like seed storage protein At2 g18540 (AUR62003182-RA), oleosin 1 (AUR62036943-RA and AUR62040213-RA), oil body-associated protein 1A (AUR62020909-RA and AUR62018510-RA), oil body-associated protein 2A (AUR62044500-RA and AUR62036611-RA), alpha-amylase (AUR62012986-RA), beta-amylase (AUR62014945-RA and AUR62015004-RA), sucrose synthase (AUR62025532-RA and AUR62008699-RA), pyruvate decarboxylase 1 (AUR62036556-RA and AUR62031145-RA), pyruvate decarboxylase 2 (AUR62010449-RA), annexin D2 (AUR62002012-RA and AUR62003790-RA), annexin D3 (AUR62037213-RA), glyceraldehyde-3-phosphate dehydrogenase, cytosolic (AUR62024167-RA), acyl-coenzyme A oxidase 2, peroxisomal (AUR62002026-RA and AUR62003772-RA), succinate dehydrogenase (ubiquinone) iron-sulfur subunit 3, mitochondrial (AUR62003783-RA), a 15.7-kDa heat shock protein, peroxisomal (AUR62018223-RA), a 17.4-kDa class III heat shock protein (AUR62001433-RA), and a 26.5-kDa heat shock protein, mitochondrial (AUR62016346-RA).

**Figure 5:**
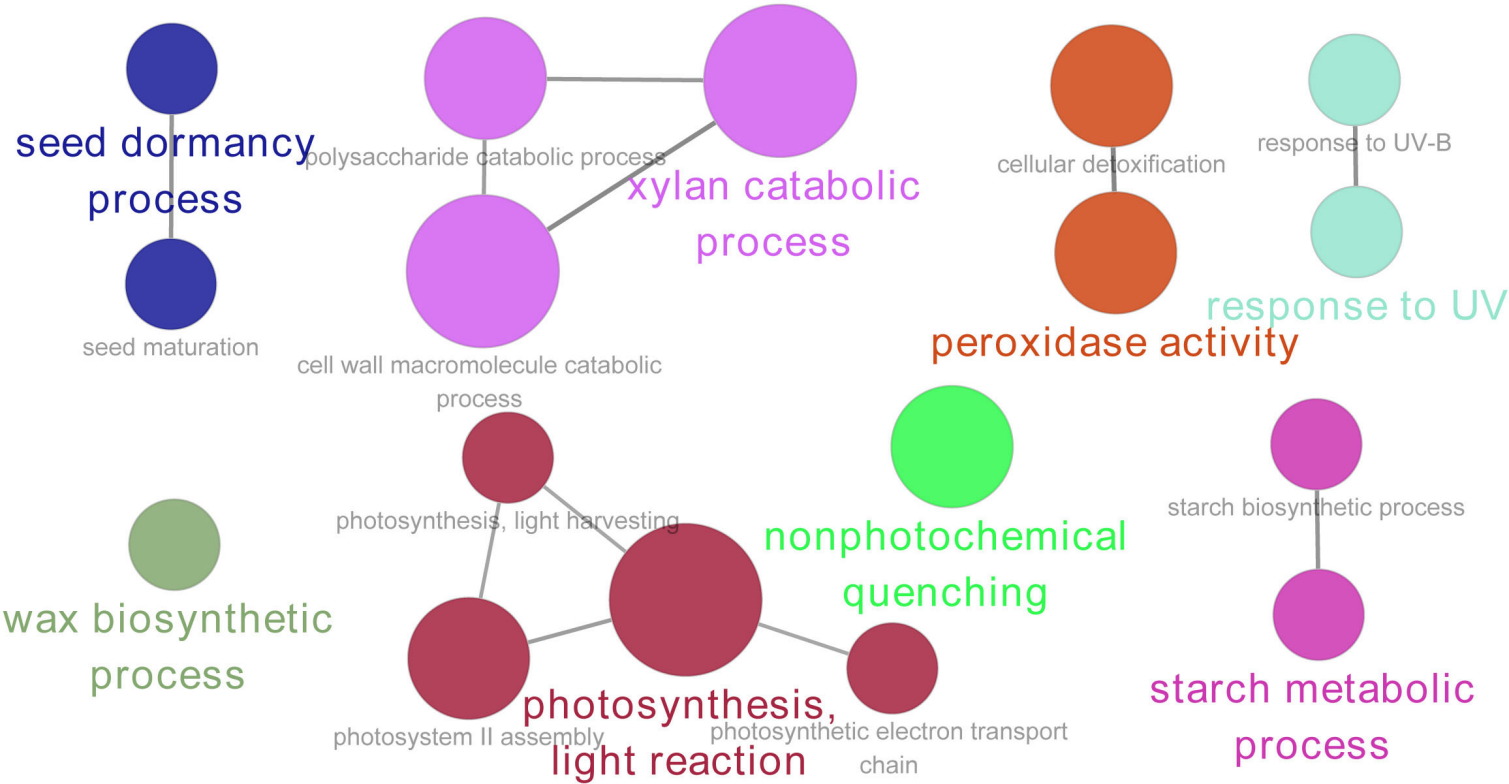
Gene Ontology (GO) enrichment (biological process) analysis of DAPs in quinoa during seed maturation. The size of the node represents the number of annotated proteins, and the color represents different GO terms (*p* < 0.05).

The GO enrichment analysis showed that the DAPs were mainly enriched in eight GO terms, including the seed dormancy process, xylan catabolic process, peroxidase activity, response to UV, wax biosynthetic process, photosynthesis light reaction, non-photochemical quenching, and starch metabolic process (**Figure 5**).

### PRM and qRT-PCR

**Figure 6:**
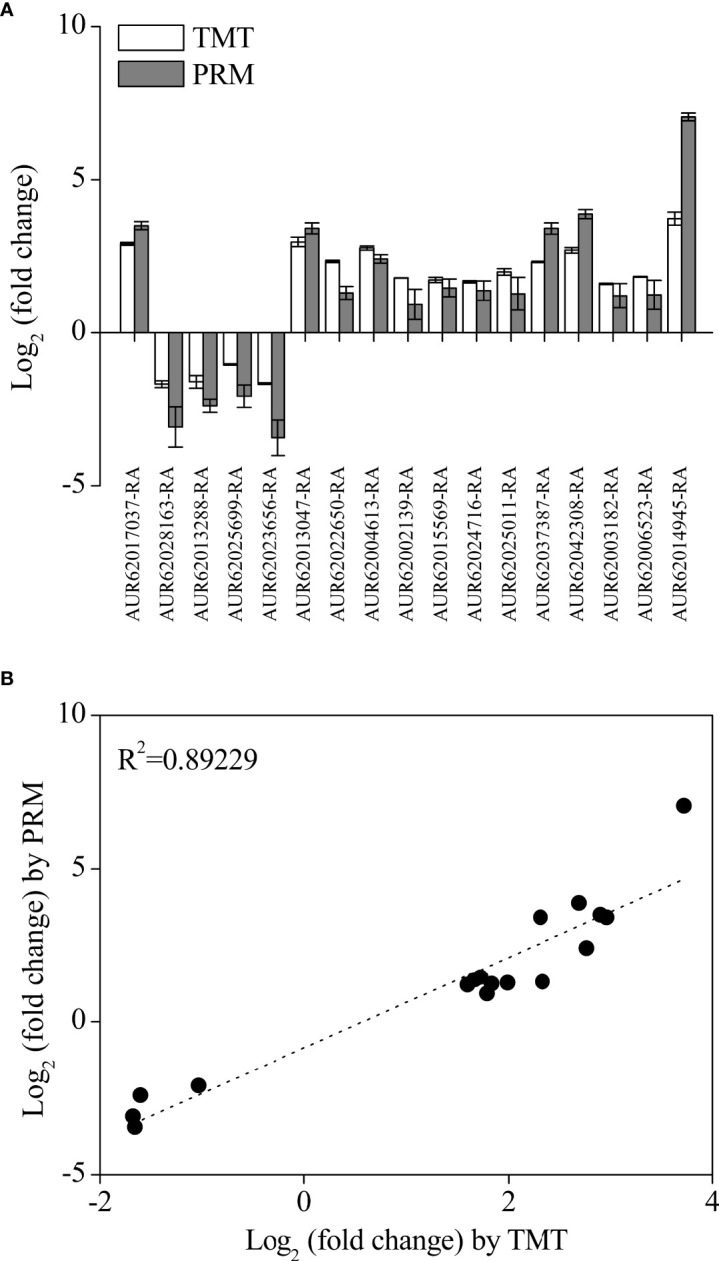
Changes in the 17 selected differentially accumulated proteins (DAPs) through tandem mass tag and parallel reaction monitoring analysis **(A)** and their correlation analysis **(B)** in quinoa from different developmental stages. The selected DAPs, including ABA-inducible protein PHV A1 (AUR62017037-RA), beta-amylase (AUR62014945-RA), 13S globulin seed storage protein 1 (AUR62002139-RA and AUR62015569-RA), 11S globulin seed storage protein 2 isoform X2 (AUR62024716-RA), vicilin-like seed storage protein At2g18540 (AUR62003182-RA), vicilin-like seed storage protein At2g28490 isoform X2 (AUR62006523-RA and AUR62025011-RA), seed biotin-containing protein SBP65 (AUR62037387-RA and AUR62042308-RA), late embryogenesis abundant protein (family protein (AUR62022650-RA and AUR62004613-RA), early-responsive to dehydration 7, chloroplastic (AUR62013047-RA), β-amyrin 28-oxidase (AUR620256 99-RA), anthocyanidin 3-O-glucosyltransferase 7 (AUR62028163-RA), bifunctional purple acid phosphatase 26 (AUR62013288-RA), and cyprosin (AUR62023656-RA). The data are presented as the mean ± SE of three replicates (*p* < 0.05).

**Figure 7:**
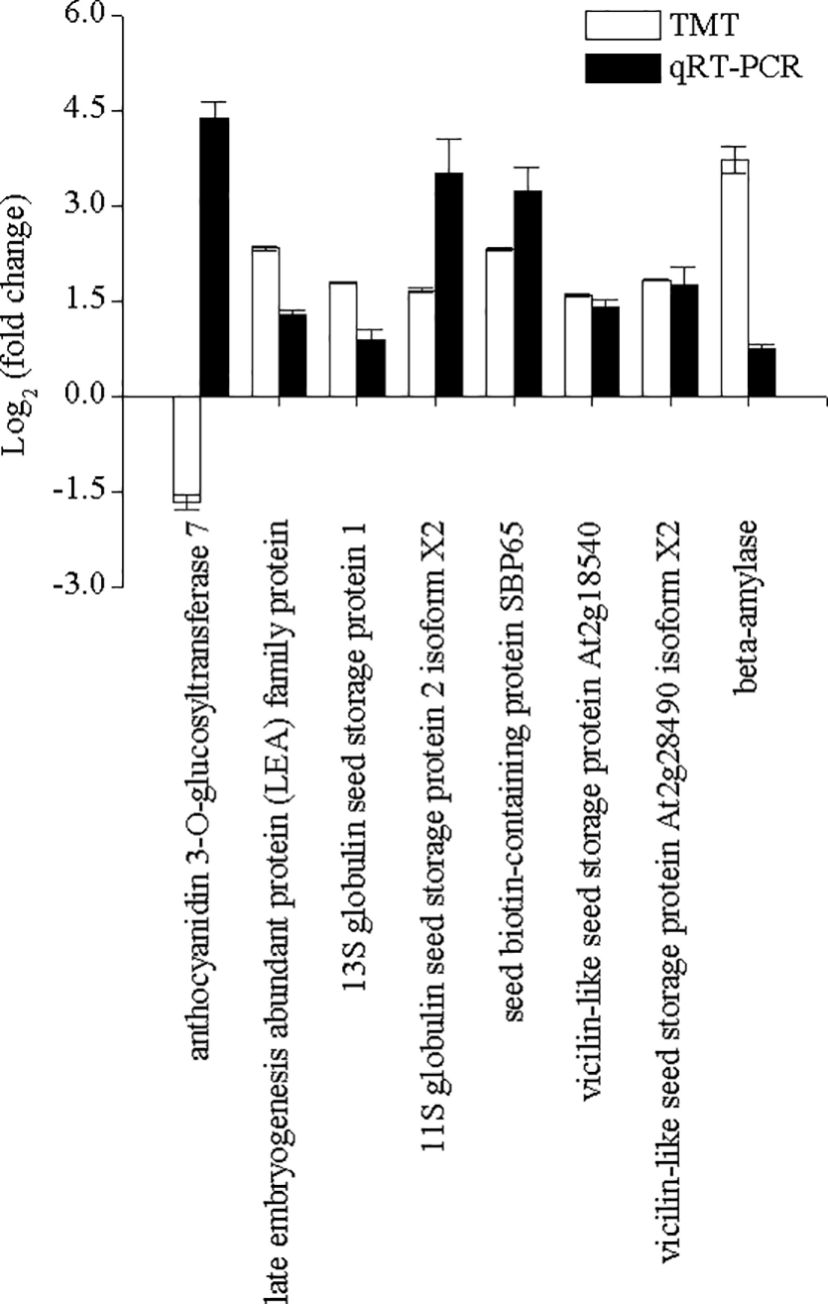
The changes in eight selected differentially accumulated proteins were determined by performing the tandem mass tag and qRT-PCR analyses in quinoa from different developmental stages. The selected DAPs included beta-amylase (AUR62014945-RA), 13S globulin seed storage protein 1 (AUR62002139-RA), 11S globulin seed storage protein 2 isoform X2 (AUR62024716-RA), vicilin-like seed storage protein At2g18540 (AUR62003182-RA), vicilin-like seed storage protein At2g28490 isoform X2 (AUR62006523-RA), seed biotin-containing protein SBP65 (AUR62037387-RA), late embryogenesis abundant protein family protein (AUR62022650-RA), and anthocyanidin 3-O-glucosyltransferase 7 (AUR62028163-RA). Tub-6 was used as the housekeeping gene. The data are presented as the mean ± SE of three replicates (*p* < 0.05).

To confirm the TMT proteomics results, 17 DAPs were selected for validation by the PRM assay (**Figure 6**). The results showed that the expression profile of all selected DAPs in the TMT assay was consistent with the PRM assay (**Figure 6A**). The high consistency (*R* ^2^ = 0.89229) between PRM and TMT quantification results validated the TMT data (**Figure 6B**). Additionally, the expression levels of eight DAPs were further validated by performing qRT-PCR. The expression levels of most DAPs were consistent with the TMT results, except for the results of anthocyanidin 3-O-glucosyltransferase 7 (AUR62028163-RA) (**Figure 7**).

## Discussion

Quinoa is an excellent crop with balanced nutrition and many adaptations. However, despite its agronomic potential, quinoa is still an underutilized crop, which can be used to enhance the global food security for a growing global population. Quinoa production suffers from many problems, such as the “green seed problem”, bitterness of saponins, nutrition accumulation, seed maturity consistency, pre-harvest sprouting, *etc*. To solve these problems, a better understanding of protein variation during quinoa seed maturation is required for genetic improvement. Therefore, protein variation in quinoa seeds during the maturation process was systematically investigated by using TMT proteomics and PRM analysis.

### Inhibition of seed photosynthesis during seed maturation

Photosynthesis is the fundamental biological process that is necessary to supply energy for plant growth and development. However, during the late stages of seed maturation, photosynthesis in seeds is inhibited due to the disintegration of the photosynthetic apparatus and chlorophyll degradation (Smolikova et al., 2011; Smolikova and Medvedev, 2016). It was suggested that photosynthesis is unnecessary for the last stage of seed maturation—for example, during the development of *Arabidopsis*, siliques grown in the dark exhibited normal seed development and produced viable mature seeds (Kim et al., 2009; Liu et al., 2017). Allorent et al. (2015) reported that the inhibition of embryonic photosynthesis in *Arabidopsis* did not affect the final lipid and protein food stores. On the contrary, the incomplete degradation of chlorophyll leads to “green seed problem” in crops and might reduce the yield and tolerance to various types of stress (Jalink et al., 1998; Chung et al., 2006; Smolikova et al., 2011). Therefore, inhibition of photosynthesis is required for seed maturation. In this study, the analysis of the morphological characteristics showed that the color of the palea and the episperm of quinoa seeds turned yellow or orange, implying reduction of photosynthesis and variation in the pigment components of quinoa seeds (**Table 1**). The DAPs involved in photosynthesis were identified by proteomics analysis, and most DAPs were downregulated during quinoa seed maturation, indicating the inhibition of photosynthesis in quinoa seeds (**Figures 3A, B** and **Supplementary Table S1**). In addition, the GO enrichment analysis showed that these proteins were enriched in light reaction, light harvesting, photosystem II assembly, and photosynthetic electron transport chain (**Figure 5**). Based on these results, it was suggested that quinoa seeds reduced the expression of proteins involved in light reaction, light harvesting, photosystem II assembly, and photosynthetic electron transport chain to inhibit photosynthesis during seed maturation, thus effectively preventing the “green seed problem” during the development of quinoa seeds.

Color change is also a sign of seed maturity. Betacyanins are the major pigments present in quinoa. In the biosynthesis pathway of betacyanins, the terminal glycosylation of aglycone betanidin transfers betanidin to batanins (Isayenkova et al., 2006; Das et al., 2013). Anthocyanins and betalains are not found in the same plant species. Interestingly, some studies reported that the genes responsible for the biosynthesis of anthocyanins were identified in betalain-accumulating plants (Xu et al., 2015; Xu et al., 2016). It was hypothesized that betacyanin and anthocyanin metabolisms coexisted in earlier plants and were separated during the evolution of the plants (Das et al., 2013). The DAP AUR62028163-RA was annotated as an anthocyanidin 3-O-glucosyltransferase 7. The similarity between the 3-O-glucosyltransferase of quinoa and anthocyanin 3-O-glucosyltransferase indicated the coexistence of both pathways of pigment biosynthesis. The protein AUR62028163-RA upregulated the transfer of glucose from UDP-glucose to betalains. This indicated that the accumulation of betalains in seeds increased as quinoa seeds approached the maturation stage.

### Activation of glycolysis during seed maturation

Previous studies have reported that seeds suffer from low oxygen stress during seed maturation (Chung et al., 1997; Rolletschek et al., 2003). Rolletschek et al. (2003) found that oxygen concentration was low in legume seeds, but embryogenic photosynthesis provided the oxygen and energy required for seed respiration (Rolletschek et al., 2003). According to our proteomics analysis, the proteins involved in photosynthesis were largely downregulated during seed maturation. It would lead to low photosynthetic activity and oxygen production in quinoa seeds (**Figure 3**, **Supplementary Table S1**), implying a shortage of energy for seed development. To acclimate to anaerobic conditions and supply energy for seed development, seeds will activate anaerobic respiration (Zhou et al., 2020). The enzyme activity in glycolysis, which plays a crucial role in energy supply in plants (Toro and Pinto, 2015), was significantly induced during seed maturation. These enzymes were, namely, aldolase, glyceraldehyde-3-phosphate dehydrogenase, pyruvate decarboxylase, and alcohol dehydrogenase (Chung et al., 1997). Similar to the previous study, our proteomics data also found an accumulation of glyceraldehyde-3-phosphate dehydrogenase (AUR62024167-RA), pyruvate decarboxylase (AUR62036556-RA, AUR62031145-RA, and AUR62010449-RA), and alcohol dehydrogenase (AUR62026788-RA) during seed maturation. These results suggested that the quinoa seed enhances the accumulation of proteins involved in glycolysis to overcome the energy shortage caused by low photosynthesis during seed maturation.

### Inhibition of saponin biosynthesis during seed maturation

The DAPs AUR62025699-RA and AUR62001317-RA were annotated as β-amyrin 28-oxidase, a saponin biosynthesis enzyme. Quinoa contains 2–5% saponins in the external layers of the seeds or leaves. These saponins produce an undesirable bitter flavor (Medina-Meza et al., 2016). According to saponin content, quinoa varieties are classified as “sweet” (free or less than 0.11 g/100 g dry weight, DW) and “bitter” varieties (more than 0.11 g/100 g DW) (Vega-Galvez et al., 2010). Because of the associated bitterness and toxicity of saponins, quinoa is treated with washing, dehulling, or thermal processing to reduce the saponin levels (Gomez-Caravaca et al., 2014; Medina-Meza et al., 2016). These processes are costly and water-intensive and can also reduce the nutritional value of the seeds (Zurita-Silva et al., 2014). Therefore, the development of saponin-free lines is a major objective concerning quinoa breeding (Zurita-Silva et al., 2014). Oleanane-type triterpenes are the major saponin components found in quinoa (Medina-Meza et al., 2016). The enzyme β-amyrin 28-oxidase is essential for oleanane-type saponin biosynthesis (Han et al., 2013; Jo et al., 2017). In this study, the downregulation of β-amyrin 28-oxidase indicated the low saponin content in quinoa seeds, which made it more “sweet” (**Figure 6** and **Supplementary Table S1**).

### Accumulation of seed storage proteins during seed maturation

Our proteomics data found that four globulin seed storage proteins (AUR62024716-RA, AUR62002139-RA, AUR62015569-RA, and AUR62021514-RA), two 2S seed storage proteins (AUR62020540-RA and AUR62015663-RA), two legumins (AUR62024712-RA and AUR62011869-RA), and eight vicilins seed storage protein (AUR62003182-RA, AUR62025011-RA, AUR62006523-RA, AUR62034727-RA, AUR62016063-RA, AUR62033661-RA, AUR62028591-RA, and AUR62032318-RA) accumulated during seed maturation (**Table 2**). Previous studies have reported that the main storage proteins in quinoa seeds are globulins, legumins, and vicilins, which are richer in lysine, methionine, and cysteine than common cereals and legumes (Ruales & Nair, 1992; Burrieza et al., 2019). Of the total protein in mature seeds of quinoa, 37% of the proteins are 11S-type globulin called chenopodin, and 35% of the seed proteins are 2S seed storage proteins (Dakhili et al., 2019). 11S globulin is the predominant component of quinoa seed storage proteins (Stevens et al., 2006; Dakhili et al., 2019). These globulins are active peptides reported as having antibacterial, antidiabetic, antihypertensive, chemo-preventive, anti-tumoral, and antioxidant activities (Hu et al., 2017; Vilcacundo et al., 2017; Vilcacundo et al., 2018). These can be used as functional ingredients in the food industry (Alemayehu et al., 2015; Zhou et al., 2019). In *Brachypodium distachyon*, seed storage proteins (*i*.*e*., globulins) were found to accumulate largely during the maturation phase (Guillon et al., 2012). Consistent with the findings of another study, we also found that globulins, legumins, and vicilins accumulated during the seed maturation phase, suggesting that the quinoa seeds in stage II were approaching maturation.

Four oleosins (AUR62036943-RA, AUR62040213-RA, AUR62008167-RA, and AUR62012221-RA) were identified in this study. Oleosins modulate the size of oil bodies (Siloto et al., 2006). During the late stage of seed maturation, the oleosin content increased considerably, while the lack of oleosins affected the total lipid content (Li et al., 2009; Miquel et al., 2014). The overexpression of oleosin in rice enhances the seed lipid content (Liu et al., 2013). Oleosins can modulate the lipid content in plant seeds. In this study, the abundance of certain DAPs (oleosins) increased, which affected the lipid content in quinoa seeds during maturation.

### Regulation of lipid metabolism during seed maturation

The main role of the oil body in the plant cell is to accumulate nutrients and lipids (Raposo and Stenmark, 2007). Our proteomics results showed that several proteins involved in lipid metabolism were upregulated during quinoa seed maturation, such as oil body-associated proteins (AUR62020909-RA, AUR62018510-RA, AUR62044500-RA, and AUR62036611-RA), acyl-coenzyme A oxidase 2 (AUR62002026-RA and AUR62003772-RA), and non-specific lipid-transfer protein (AUR62029725-RA, AUR62029726-RA, AUR62006384-RA, and AUR62006386-RA). Oil body-associated protein 1, which influences the stability of oil bodies, accumulates during seed maturation but disappears after germination (Lopez-Ribera et al., 2014). The RNA interference of oil body-associated protein 1 showed low oil content in seed, suggesting a positive role of oil body-associated protein 1 in oil biosynthesis (Lopez-Ribera et al., 2014). The upregulation of oil body-associated proteins in the present study suggested that the accumulation of oil body-associated proteins might lead to the accumulation of oil in quinoa seeds (**Supplementary Table S1**). Additionally, our protein–protein interaction analysis found an interaction between oil body-associated proteins and oleosin (**Figure 4**). As we have mentioned above, oleosins can also regulate the lipid content in plant seeds. It was suggested that oleosins and oil body-associated proteins coordinate to regulate the oil content in quinoa seeds during seed maturation. During quinoa seed maturation, acyl-coenzyme A oxidase 2, which encodes an enzyme to catalyze the desaturation of long-chain acyl-CoA to 2-trans-enoyl-CoA and participates in the metabolism of long-chain fatty acids (Hooks et al., 1999), was increased. This implies the activation of long-chain fatty acid metabolism. Based on these results, we concluded that the accumulation of oil body-associated proteins, oleosins, and acyl-coenzyme A oxidase 2 can promote oil accumulation in quinoa seeds during seed maturation.

Non-specific lipid-transfer protein transfers phospholipids across membranes and might function in wax or cutin deposition in the cell wall. Boutrot et al., (2005) found that, during seed maturation, non-specific lipid-transfer protein 9.1a accumulated. Consistent with the findings of the previous study, we also found the accumulation of non-specific lipid-transfer protein during quinoa seed maturation, suggesting that non-specific lipid-transfer protein might play a role in quinoa seed maturation.

### DAPs in response to seed desiccation during seed maturation

During seed maturation, seed desiccation is the terminal event in embryogenesis, and variation in carbohydrate metabolism occurs. In response to seed desiccation, some stress response proteins were induced—for example, aldose reductase, which catalyzes the NADPH-dependent reduction of many carbonyl-containing compounds to their corresponding alcohols, was only found to accumulate during the seed maturation stage (Sree et al., 2000). Bartels reported that aldose reductase plays an important role in the synthesis of osmolytes in response to seed desiccation (Bartels et al., 1991). We also found the significant upregulation of aldose reductase (AUR62006286-RA, AUR62026335-RA, AUR62006284-RA, and AUR62026333-RA) during quinoa seed maturation. This indicated that aldose reductase can enhance the desiccation tolerance in quinoa during seed maturation. Besides this, early-responsive to dehydration proteins (ERDs) were found to regulate seed development and germination in *Arabidopsis* (Kim and Nam, 2010). Rai et al. (2012) reported that the transcriptional levels of ERDs rapidly increased under dehydration conditions and various abiotic stresses Rai et al. (2012). Moreover, ERDs were also proved to enhance drought tolerance by acting as sugar transporter during sugar biosynthesis, which protected plants from drought stress by regulating the osmotic balance (Rizhsky et al., 2004; Pertl-Obermeyer et al., 2016). In the present study, the upregulation of ERDs (AUR62010055-RA, AUR62013047-RA, and AUR62012323-RA) indicated that quinoa protects its seeds from desiccation by activating the accumulation of ERDs and regulating sugar biosynthesis during seed maturation.

The proteins AUR62022650-RA and AUR62004613-RA were annotated as late embryogenesis abundant (LEA) proteins. LEA proteins are involved in ABA-mediated stress responses (Galau et al., 1986). Seeds suffer from desiccation during the maturation phase due to the accumulation of high levels of LEA proteins (Avelange-Macherel et al., 2015; Saucedo et al., 2017). It was hypothesized that LEAs might have been evolutionarily selected to adopt diversified conditions driven by variations in their cellular environment (Mariana et al., 2019). The content of two LEAs (AUR62022650-RA and AUR62004613-RA) was found to be high. An increase in the content of 11S globulins and 2S albumin was also found, which was consistent with an increase in seed desiccation during the maturation of quinoa.

### Maintaining the seed dormancy of quinoa during seed maturation

Pre-harvest sprouting is cut-short dormancy or dormancy loss, and abnormal generation is a severe problem in quinoa production (**Supplementary Figure S1**). As an evolutionary strategy, seed dormancy inhibits seed germination during seed storage. The process from dormancy to germination includes three stages, namely: the primary dormancy during seed maturation, the gradual loss of seed dormancy in a subsequent period of seed storage (so-called after-ripening), and, finally, the approach to a non-dormant state (Graeber et al., 2012; Ne´e et al., 2017). Cyprosin, a member of the aspartic proteinase family, is involved in the dormancy, viability, and germination of seeds (Janek et al., 2016; Shen et al., 2018). The DAP AUR62023656-RA was identified as a downregulated cyprosin, implying that the degradation of cyprosin promoted seed dormancy.

AUR62037914-RA, AUR62040165-RA, and AUR62014787-RA were annotated as seed biotin-containing protein SBP65 (seed biotinylated protein of 65 kDa of apparent molecular mass). The proteins are biotin-dependent carboxylases, which play key roles in basic metabolism in most plants (Dehaye et al., 1997). The biotinylated proteins, devoid of any carboxylase activity, were characterized by many physiological and molecular features with LEA proteins in peas (Dehaye et al., 1997). These peculiar proteins localize to the cytosol of embryonic cells and might behave as a scavenger or a sink of free biotin during the late stages of embryo development. They are rapidly degraded during germination (Duval et al., 1994, Dehaye et al., 1997; Wang et al., 2012). Biotin plays a key role during seedling establishment from immature embryos, and ABA can induce the expression of proteins (Dehaye et al., 1997). The report indicated that the abundance of these proteins increased with seed maturity. Quinoa probably evolved a mechanism to prevent pre-harvest sprouting. Further investigation might help prevent pre-harvest sprouting in quinoa. This study helps to understand the role of these proteins in the embryonic development of higher plants.

## Conclusions

Seed maturation is an essential stage in the plant life cycle, especially for economic seed crops such as quinoa. In this study, TMT proteomics analysis of quinoa during seed maturation was performed. The results showed that the DAPs involved in photosynthesis were mostly downregulated, implying a low photosynthetic activity and a shortage of energy supply from photosynthesis during seed maturation. To overcome the shortage of energy, several DAPs involved in glycolysis, such as glyceraldehyde-3-phosphate dehydrogenase, pyruvate decarboxylase, and alcohol dehydrogenase, were upregulated to provide energy for seed maturation conversion. Besides this, the content of storage proteins, such as globulins, legumins, vicilins, and oleosin, increased significantly during quinoa seed maturation, while oleosin, oil body-associated proteins, and acyl-coenzyme A oxidase 2 could enhance oil accumulation in quinoa seed. The early-responsive to dehydration protein and late embryogenesis abundant proteins were also induced to protect the quinoa seeds from dehydration. In addition, the downregulation of β-amyrin 28-oxidase might lead to a low saponin content in quinoa seeds, making quinoa “sweet”. The variation in cyprosin and seed biotin-containing pwrotein SBP65 suggested the promotion of seed dormancy during quinoa seed maturation. This study enhanced our understanding of the biological and physiological characteristics of seed maturation in quinoa. Our findings might be an important first step toward the genetic improvement of tropical quinoa.

## Data availability statement

The original contributions presented in the study are publicly available. This data can be found here: ProteomeXchange, PXD034885 https://www.ebi.ac.uk/pride/archive/projects/PXD034885.

## Author contributions

Z-JS: methodology, software, investigation, formal analysis, writing—original draft, and visualization. S-XX: investigation, conceptualization, methodology, supervision, writing—review and editing, project and administration, and funding acquisition. Q-YH: methodology, software, investigation, and formal analysis. Z-YL: resources and writing—review and editing. Y-DX: software and writing—review and editing. C-SL: methodology and writing—review and editing. Y-JH: formal analysis and writing—review and editing. All authors contributed to the article and approved the submitted version.

## Funding

This work was supported by the National Natural Science Foundation of China (32071786) and the Xiamen Science and Technology Project (grant numbers: 3502Z20172015, 3502Z20194503, 3502Z20194504, and 3502Z20211006-20210915).

## Conflict of interest

The authors declare that the research was conducted in the absence of any commercial or financial relationships that could be construed as a potential conflict of interest.

## Publisher’s note

All claims expressed in this article are solely those of the authors and do not necessarily represent those of their affiliated organizations, or those of the publisher, the editors and the reviewers. Any product that may be evaluated in this article, or claim that may be made by its manufacturer, is not guaranteed or endorsed by the publisher.

## Abbreviations

ABA, abscisic acid; AGC, automatic gain control; BAM, β-amylase; BCA, bicinchoninic acid; DAPs, differentially accumulated proteins; ERD, early-responsive to dehydration; GO, Gene Ontology; KEGG, Kyoto Encyclopedia of Genes and Genomes; LEA, late embryogenesis abundant proteins; PRM, parallel reaction monitoring; TEAB, triethylamonium bicarbonate; TMT, tandem mass tag; UDP, uridinediphosphate; UGT, UDP-glycosyltransferase.
