# Supplementary figures and images for: TMT proteomics analysis of a pseudocereal crop, quinoa (Chenopodium quinoa Willd.), during seed maturation
Source: Front Plant Sci. 2022 Nov 8;13:975073. doi: 10.3389/fpls.2022.975073 (PMC9678934; doi:10.3389/fpls.2022.975073)

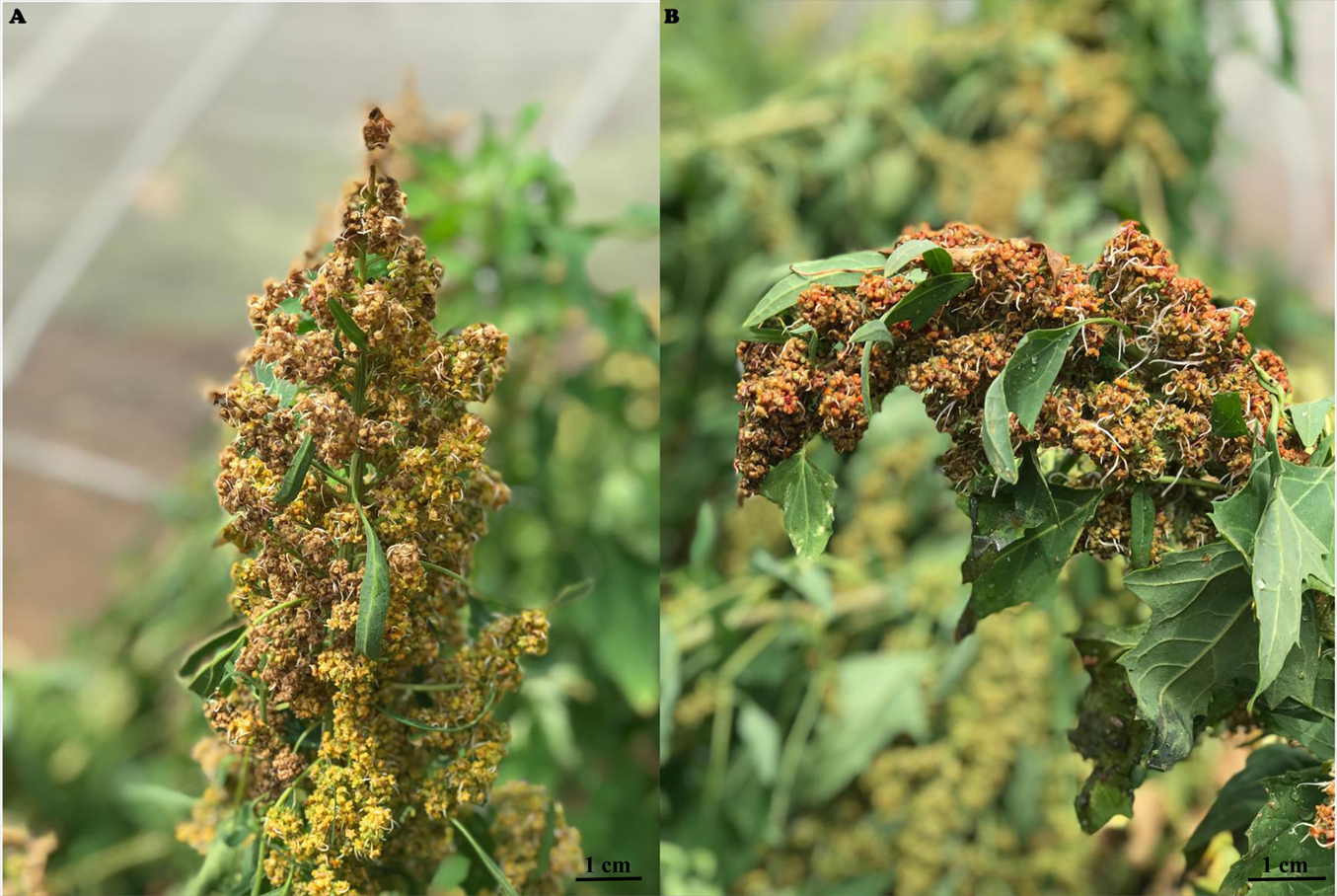

Supplement: Supplementary file 1 [file Image_1.tif]
