# Supplementary material for: TMT proteomics analysis of a pseudocereal crop, quinoa (Chenopodium quinoa Willd.), during seed maturation
Source: Front Plant Sci. 2022 Nov 8;13:975073. doi: 10.3389/fpls.2022.975073 (PMC9678934; doi:10.3389/fpls.2022.975073)
Supplement: Supplementary file 2 [file Table_1.docx]

**Supplementary Table S1.** Differentially accumulated proteins (DAPs) identified from mature seed (MS) and immature seed (IS) of *Chenopodium quinoa* willd

| **Accession No.** | **Protein description** | **MW (kDa)** | **Score** | **Coverage (%)** | **Peptides** | **Regulated Type** | **P-value** |
| --- | --- | --- | --- | --- | --- | --- | --- |
| **Photosynthesis** | |  |  |  |  |  |  |
| AUR62037524-RA | ATP-dependent zinc metalloprotease FTSH 6, chloroplastic | 74.585 | 1.2701 | 5.9 | 3 | Down | 1.98285E-05 |
| AUR62036924-RA | Calcium sensing receptor, chloroplastic | 42.474 | 35.878 | 21 | 7 | Down | 0.00003684 |
| AUR62014571-RA | NADH-ubiquinone reductase complex 1 MLRQ subunit-like protein | 9.9293 | 15.385 | 22.5 | 2 | Down | 0.00004061 |
| AUR62015903-RA | Cell division protein FtsZ homolog 1, chloroplastic | 45.526 | 47.589 | 18.2 | 6 | Down | 0.0046024 |
| AUR62020320-RA | Cell division protein FtsZ homolog 1, chloroplastic | 38.391 | 33.715 | 16.2 | 5 | Down | 0.00023728 |
| AUR62013862-RA | Plastocyanin | 10.691 | 42.131 | 40.6 | 2 | Down | 1.68314E-05 |
| AUR62029310-RA | Chlorophyll a-b binding protein 13, chloroplastic | 28.5 | 13.567 | 9.5 | 2 | Down | 0.00068382 |
| AUR62040811-RA | Photosystem I chlorophyll a/b-binding protein 3-1, chloroplastic | 29.524 | 73.97 | 32.1 | 7 | Down | 0.000043145 |
| AUR62008430-RA | Chlorophyll a-b binding protein 4, chloroplastic | 27.966 | 8.224 | 4.7 | 1 | Down | 1.6044E-07 |
| AUR62017505-RA | Chlorophyll a-b binding protein 5, chloroplastic | 28.524 | 5.7633 | 15.9 | 4 | Down | 0.00044141 |
| AUR62031289-RA | Chlorophyll a-b binding protein CP24 10A, chloroplastic | 27.744 | 24.125 | 13 | 3 | Down | 0.0053243 |
| AUR62027370-RA | Chlorophyll a-b binding protein CP24 10A, chloroplastic-like | 27.744 | 23.874 | 24.2 | 6 | Down | 5.8553E-07 |
| AUR62022613-RA | Chlorophyll a-b binding protein CP26, chloroplastic | 26.155 | 7.3575 | 32.4 | 6 | Down | 1.9386E-06 |
| AUR62004407-RA | chlorophyll a-b binding protein CP26, chloroplastic-like | 26.225 | 56.696 | 33.1 | 6 | Down | 0.00023823 |
| AUR62041844-RA | Chlorophyll a-b binding protein CP29.1, chloroplastic | 31.122 | 11.832 | 21 | 5 | Down | 0.000119656 |
| AUR62007188-RA | Chlorophyll a-b binding protein CP29.3, chloroplastic | 31.379 | 52.451 | 44.3 | 10 | Down | 0.000039823 |
| AUR62009677-RA | Chlorophyll a-b binding protein, chloroplastic-like | 28.326 | 21.474 | 22.1 | 5 | Down | 5.3408E-07 |
| AUR62030254-RA | Chlorophyll a-b binding protein, chloroplastic-like | 28.316 | 6.2305 | 17.2 | 4 | Down | 0.00026346 |
| AUR62018842-RA | Light-harvesting complex-like protein 3 isotype 2, chloroplastic | 30.547 | 19.78 | 10.3 | 3 | Down | 0.0095207 |
| AUR62007164-RA | Light-harvesting complex-like protein 3 isotype 2, chloroplastic | 28.568 | 1.883 | 7.5 | 2 | Down | 0.000137591 |
| AUR62034898-RA | Light-harvesting complex-like protein OHP2, chloroplastic | 8.2423 | 2.8602 | 31.1 | 2 | Down | 3.1098E-06 |
| AUR62033720-RA | Cytochrome b6-f complex iron-sulfur subunit, chloroplastic | 24.177 | 9.9946 | 18.3 | 3 | Down | 0.00066241 |
| AUR62025154-RA | Cytochrome b6-f complex iron-sulfur subunit, chloroplastic | 24.202 | 47.269 | 24.3 | 4 | Down | 1.52658E-05 |
| AUR62000091-RA | Oxygen-evolving enhancer protein 1, chloroplastic | 35.139 | 175.73 | 49.2 | 16 | Down | 0.0003197 |
| AUR62017838-RA | Oxygen-evolving enhancer protein 2, chloroplastic | 29.289 | 68.55 | 33.9 | 9 | Down | 0.0028775 |
| AUR62003524-RA | Oxygen-evolving enhancer protein 2, chloroplastic | 29.432 | 2.9309 | 33.9 | 9 | Down | 0.00140025 |
| AUR62012582-RA | Oxygen-evolving enhancer protein 3, chloroplastic | 24.875 | 3.5478 | 38.2 | 8 | Down | 0.003182 |
| AUR62044485-RA | Oxygen-evolving enhancer protein 3, chloroplastic | 24.588 | 74.936 | 44.5 | 10 | Down | 3.9786E-06 |
| AUR62007866-RA | Photosystem I reaction center subunit II, chloroplastic-like | 23 | 57.922 | 43.6 | 7 | Down | 0.000062941 |
| AUR62003053-RA | Photosystem I reaction center subunit II, chloroplastic-like | 23.026 | 9.7498 | 43.6 | 7 | Down | 0.000079931 |
| AUR62025395-RA | Photosystem I reaction center subunit IV, chloroplastic | 11.561 | 28.152 | 22.6 | 2 | Down | 3.5237E-06 |
| AUR62038135-RA | Photosystem I reaction center subunit psaK, chloroplastic | 13.148 | 3.9574 | 6.9 | 1 | Down | 0.000104745 |
| AUR62006659-RA | Photosystem I reaction center subunit XI, chloroplastic | 22.944 | 27.849 | 17.1 | 3 | Down | 0.000041013 |
| AUR62022575-RA | Photosystem II 10 kDa polypeptide, chloroplastic | 14.442 | 4.278 | 18.6 | 3 | Down | 0.003177 |
| AUR62041946-RA | Photosystem II repair protein PSB27-H1, chloroplastic | 19.177 | 3.5739 | 18 | 3 | Down | 0.000057063 |
| AUR62036627-RA | Photosystem I P700 chlorophyll a apoprotein A1 (chloroplast) | 83.46 | 13.53 | 7.3 | 7 | Down | 0.000024391 |
| AUR62030860-RA | Photosystem I reaction center subunit N | 15.065 | 5.8089 | 21.5 | 3 | Down | 0.0035608 |
| AUR62024084-RA | Photosystem II CP43 chlorophyll apoprotein | 64.188 | 51.107 | 17.1 | 9 | Down | 0.000095007 |
| AUR62039871-RA | Photosystem II protein D2 (chloroplast) | 56.408 | 31.086 | 9 | 3 | Down | 1.71842E-05 |
| AUR62024096-RA | PsbP domain-containing protein 3, chloroplastic isoform X1 | 28.684 | 4.2971 | 9.2 | 2 | Down | 1.80875E-05 |
| AUR62002775-RA | psbB gene product (chloroplast) | 40.642 | 45.027 | 17.6 | 5 | Down | 0.00022137 |
| AUR62012129-RA | Ribulose bisphosphate carboxylase/oxygenase activase, chloroplastic isoform X1 | 51.753 | 155.15 | 46.7 | 19 | Down | 0.00175972 |
| AUR62026974-RA | Alpha carbonic anhydrase 7 | 32.321 | 51.951 | 10.2 | 5 | Down | 0.0006044 |
| AUR62020102-RA | Thylakoid soluble phosphoprotein TSP9 protein | 10.785 | 18.153 | 23.5 | 3 | Down | 5.5097E-07 |
| AUR62042480-RA | Protochlorophyllide reductase | 36.893 | 28.349 | 22.4 | 9 | Down | 0.00005944 |
| AUR62008423-RA | Geranylgeranyl diphosphate reductase, chloroplastic | 50.83 | 34.06 | 15 | 6 | Down | 4.3031E-07 |
| AUR62039856-RA | Glucan endo-1,3-beta-glucosidase | 34.73 | 20.378 | 17.1 | 4 | Down | 0.000061113 |
| AUR62039854-RA | Glucan endo-1,3-beta-glucosidase isoform X1 | 37.038 | 4.4932 | 12 | 4 | Down | 0.00058493 |
| AUR62039855-RA | Glucan endo-1,3-beta-glucosidase-like | 43.311 | 4.3736 | 5.3 | 2 | Down | 0.000198462 |
| AUR62041208-RA | Glutamine synthetase leaf isozyme, chloroplastic | 47.266 | 77.496 | 25.3 | 9 | Down | 0.00031847 |
| AUR62004874-RA | Rubisco accumulation factor 1.1, chloroplastic | 37.951 | 3.4949 | 9.3 | 3 | Up | 0.000164143 |
| AUR62037607-RA | Rubisco accumulation factor 1.1, chloroplastic | 50.646 | 6.1023 | 11.3 | 4 | Up | 0.000104546 |
| AUR62022174-RA | RuBisCO large subunit-binding protein subunit beta, | 65.124 | 54.746 | 24.3 | 13 | Up | 0.0114358 |
| **Carbohydrate metabolism** | |  |  |  |  |  |  |
| AUR62015518-RA | Beta-fructofuranosidase, soluble isoenzyme I | 71.245 | 7.763 | 5.6 | 3 | Down | 0.00087766 |
| AUR62025637-RA | Acylpyruvase FAHD1, mitochondrial | 23.29 | 34.347 | 22.7 | 4 | Down | 0.000040382 |
| AUR62023226-RA | Probable fructokinase-6, chloroplastic | 42.45 | 12.567 | 16.3 | 6 | Down | 0.000059874 |
| AUR62031822-RA | Alcohol dehydrogenase 3 | 41.4 | 2.2952 | 16 | 6 | Down | 0.0028962 |
| AUR62031595-RA | NADP-dependent glyceraldehyde-3-phosphate dehydrogenase | 53.244 | 50.716 | 33.1 | 13 | Down | 0.00019517 |
| AUR62009235-RA | NADP-dependent malic enzyme | 68.09 | 32.747 | 17 | 10 | Down | 0.00076046 |
| AUR62022202-RA | NADP-dependent malic enzyme | 71.71 | 14.119 | 9.3 | 6 | Down | 0.000038625 |
| AUR62016505-RA | Malonate--CoA ligase isoform X1 | 58.548 | 30.413 | 10.5 | 4 | Down | 0.00089842 |
| AUR62018527-RA | Xyloglucan endotransglucosylase/hydrolase protein 24-like | 32.695 | 37.116 | 14.8 | 4 | Down | 0.000024716 |
| AUR62031797-RA | D-3-phosphoglycerate dehydrogenase 1, chloroplastic | 44.492 | 3.7873 | 12.9 | 5 | Down | 0.00034195 |
| AUR62016823-RA | Fructose-bisphosphate aldolase 1, chloroplastic | 43.069 | 92.64 | 47 | 15 | Down | 0.000038223 |
| AUR62022835-RA | Sedoheptulose-1,7-bisphosphatase, chloroplastic | 42.223 | 67.349 | 30.2 | 10 | Down | 0.000043336 |
| AUR62042589-RA | Glyceraldehyde-3-phosphate dehydrogenase B, chloroplastic | 41.056 | 322.83 | 28.5 | 10 | Down | 4.8129E-06 |
| AUR62043666-RA | Glycerate dehydrogenase HPR, peroxisomal | 42.008 | 60.571 | 39.1 | 12 | Down | 1.56991E-05 |
| AUR62024904-RA | Cellulose synthase A catalytic subunit 1 [UDP-forming] | 117.69 | 4.4357 | 2.9 | 3 | Down | 0.000099902 |
| AUR62021274-RA | Probable carboxylesterase 120 | 37.912 | 1.4872 | 2.4 | 1 | Down | 0.0154041 |
| AUR62004255-RA | Probable carboxylesterase 8 | 37.553 | 39.433 | 28.2 | 7 | Down | 0.000035142 |
| AUR62023862-RA | Probable fructokinase-4 | 36.102 | 28.167 | 36.3 | 9 | Down | 0.00087973 |
| AUR62022577-RA | Probable glycerol-3-phosphate acyltransferase 8 | 56.094 | 16.683 | 10.2 | 5 | Down | 0.000024546 |
| AUR62003633-RA | Probable mannitol dehydrogenase | 39.082 | 35.128 | 14.1 | 4 | Down | 4.4189E-06 |
| AUR62003695-RA | Probable mannitol dehydrogenase | 39.325 | 14.995 | 14.2 | 5 | Down | 0.00093913 |
| AUR62017942-RA | Probable mannitol dehydrogenase | 39.142 | 35.082 | 17.4 | 6 | Down | 2.9266E-07 |
| AUR62027775-RA | Probable pectin methyltransferase QUA2 isoform X1 | 79.148 | 5.8739 | 3.4 | 2 | Down | 0.00044493 |
| AUR62024669-RA | Probable beta-D-xylosidase 2 | 82.815 | 0.998 | 6.9 | 5 | Down | 0.00055549 |
| AUR62038289-RA | Glucomannan 4-beta-mannosyltransferase 2-like | 62.187 | 3.3851 | 2 | 1 | Down | 4.8494E-06 |
| AUR62025699-RA | Beta-amyrin 28-oxidase | 54.985 | 31.336 | 19.7 | 8 | Down | 1.58917E-05 |
| AUR62001317-RA | Beta-amyrin 28-oxidase | 54.693 | 3.4734 | 18.2 | 7 | Down | 0.00081673 |
| AUR62006286-RA | Aldose reductase | 36.295 | 57.069 | 44.1 | 16 | Up | 0.000021349 |
| AUR62026335-RA | Aldose reductase | 36.454 | 8.446 | 39.1 | 14 | Up | 3.3513E-06 |
| AUR62006284-RA | Aldose reductase-like | 37.217 | 18.987 | 43.5 | 15 | Up | 2.4212E-07 |
| AUR62026333-RA | Aldose reductase-like | 37.165 | 66.842 | 43.2 | 15 | Up | 6.4958E-08 |
| AUR62036556-RA | Pyruvate decarboxylase 1 | 61.796 | 32.745 | 16.5 | 8 | Up | 1.84508E-05 |
| AUR62031145-RA | Pyruvate decarboxylase 1 | 61.767 | 0.98925 | 14.5 | 7 | Up | 0.000157895 |
| AUR62010449-RA | Pyruvate decarboxylase 2 | 61.085 | 74.38 | 32.8 | 16 | Up | 2.7853E-06 |
| AUR62026788-RA | Alcohol dehydrogenase 3 | 41.252 | 13.143 | 21.1 | 9 | Up | 0.000077598 |
| AUR62024167-RA | Glyceraldehyde-3-phosphate dehydrogenase, cytosolic | 35.962 | 9.723 | 39.9 | 12 | Up | 1.55278E-05 |
| AUR62006360-RA | Pullulanase 1, chloroplastic | 101.96 | 179.38 | 33.6 | 26 | Up | 1.86711E-05 |
| AUR62003783-RA | Succinate dehydrogenase [ubiquinone] iron-sulfur subunit 3, mitochondrial | 35.838 | 3.7418 | 9.5 | 3 | Up | 1.2039E-06 |
| AUR62031439-RA | Alpha-glucosidase | 75.932 | 20.496 | 15.9 | 11 | Up | 0.00041798 |
| AUR62021100-RA | Alpha-glucosidase | 131.46 | 72.569 | 13.3 | 16 | Up | 1.2895E-06 |
| AUR62000840-RA | Glucose and ribitol dehydrogenase | 32.849 | 183.64 | 58 | 16 | Up | 0.000080239 |
| AUR62005133-RA | Glucose and ribitol dehydrogenase | 31.917 | 15.024 | 52.2 | 14 | Up | 0.000063616 |
| AUR62005134-RA | Glucose and ribitol dehydrogenase homolog 1 | 31.793 | 85.85 | 45.1 | 15 | Up | 1.62224E-05 |
| AUR62000839-RA | Glucose and ribitol dehydrogenase homolog 1 | 35.589 | 25.755 | 33.7 | 13 | Up | 0.000076339 |
| AUR62012986-RA | Alpha-amylase | 44.279 | 48.29 | 24.7 | 8 | Up | 0.000035577 |
| AUR62014945-RA | Beta-amylase | 56.796 | 121.61 | 40.1 | 17 | Up | 0.0027434 |
| AUR62015004-RA | Beta-amylase | 57.033 | 13.4 | 21 | 9 | Up | 0.000104462 |
| AUR62016137-RA | Beta-D-xylosidase 1 | 83.405 | 18.625 | 12.5 | 8 | Up | 0.000038079 |
| AUR62025909-RA | Beta-glucosidase 13 | 63.598 | 82.412 | 26.4 | 17 | Up | 0.00038218 |
| AUR62032862-RA | ATP-dependent 6-phosphofructokinase 2 | 52.049 | 2.8317 | 13.4 | 7 | Up | 0.000058248 |
| AUR62005870-RA | ATP-dependent 6-phosphofructokinase 2 | 52.445 | 12.793 | 15.4 | 8 | Up | 0.00006177 |
| AUR62025532-RA | Sucrose synthase | 90.556 | 2.7273 | 13 | 10 | Up | 0.000059223 |
| AUR62008699-RA | Sucrose synthase | 93.971 | 20.325 | 13.9 | 11 | Up | 0.00027716 |
| AUR62027506-RA | Putative beta-galactosidase | 63.322 | 21.981 | 7.2 | 3 | Up | 0.0029579 |
| **Lipid metabolism** | |  |  |  |  |  |  |
| AUR62014540-RA | Allene oxide cyclase, chloroplastic isoform X2 | 27.21 | 11.222 | 15.7 | 3 | Down | 0.042865 |
| AUR62017459-RA | Acetyl-coenzyme A synthetase, chloroplastic/glyoxysomal | 76.73 | 91.924 | 19 | 10 | Down | 0.000104183 |
| AUR62000076-RA | Linoleate 13S-lipoxygenase 2-1, chloroplastic | 99.162 | 36.81 | 28 | 21 | Down | 0.000104189 |
| AUR62019248-RA | Linoleate 13S-lipoxygenase 2-1, chloroplastic | 103.38 | 154.64 | 34.9 | 29 | Down | 0.000059323 |
| AUR62024149-RA | Lipoxygenase 6, chloroplastic | 103.13 | 1.5833 | 8 | 6 | Down | 0.0058618 |
| AUR62025737-RA | GDSL esterase/lipase At3g48460 | 43.262 | 8.4439 | 5.8 | 2 | Down | 0.0032977 |
| AUR62035846-RA | GDSL esterase/lipase At4g18970 | 38.846 | 30.739 | 13 | 4 | Down | 0.0121375 |
| AUR62006994-RA | GDSL esterase/lipase At5g33370 | 40.4 | 53.94 | 20.2 | 5 | Down | 0.00006368 |
| AUR62035844-RA | GDSL esterase/lipase At5g45670 | 40.394 | 52.471 | 16.3 | 5 | Down | 0.000079703 |
| AUR62018263-RA | GDSL esterase/lipase EXL3 | 33.105 | 4.0426 | 3.6 | 1 | Down | 0.000023993 |
| AUR62011760-RA | Long chain acyl-CoA synthetase 1 | 69.788 | 37.657 | 16.2 | 9 | Down | 0.0025011 |
| AUR62011387-RA | Long chain acyl-CoA synthetase 2 | 95.761 | 31.157 | 8.3 | 6 | Down | 0.0040803 |
| AUR62016673-RA | Long chain acyl-CoA synthetase 2 | 73.579 | 19.164 | 10.9 | 6 | Down | 0.000021511 |
| AUR62016551-RA | Oleoyl-acyl carrier protein thioesterase 1, chloroplastic | 42.261 | 20.931 | 15.5 | 5 | Down | 6.4454E-07 |
| AUR62013350-RA | Phospholipase D alpha 1 | 91.138 | 60.859 | 23.4 | 14 | Down | 0.00085698 |
| AUR62015373-RA | Delta(12)-fatty-acid desaturase FAD2 | 44.096 | 6.4682 | 6.8 | 2 | Down | 0.00101971 |
| AUR62006617-RA | Stearoyl-[acyl-carrier-protein] 9-desaturase, chloroplastic | 45.398 | 49.586 | 19.8 | 8 | Down | 0.000039454 |
| AUR62036718-RA | 24-methylenesterol C-methyltransferase 2 | 76.166 | 18.752 | 8.3 | 6 | Down | 0.00005866 |
| AUR62013876-RA | 24-methylenesterol C-methyltransferase 2 | 40.32 | 32.421 | 17.7 | 7 | Down | 1.4575E-06 |
| AUR62023656-RA | Cyprosin | 55.534 | 16.059 | 14.4 | 8 | Down | 0.000016448 |
| AUR62007416-RA | Acyl-CoA-binding protein | 9.4555 | 3.0079 | 56.5 | 4 | Down | 0.030521 |
| AUR62000623-RA | Acyl-coenzyme A thioesterase 13 | 16.788 | 2.3877 | 7.1 | 1 | Down | 0.0038567 |
| AUR62010579-RA | Hydroxymethylglutaryl-CoA synthase | 52.042 | 17.794 | 10.2 | 4 | Down | 1.2263E-06 |
| AUR62002026-RA | Acyl-coenzyme A oxidase 2, peroxisomal | 80.269 | 1.7501 | 14.3 | 9 | Up | 0.000036838 |
| AUR62003772-RA | Acyl-coenzyme A oxidase 2, peroxisomal | 71.449 | 28.61 | 16 | 9 | Up | 0.0071195 |
| AUR62029725-RA | Non-specific lipid-transfer protein | 22.01 | 15.064 | 24.4 | 5 | Up | 2.6471E-07 |
| AUR62029726-RA | Non-specific lipid-transfer protein | 12.878 | 12.991 | 42.1 | 4 | Up | 0.00013731 |
| AUR62006384-RA | Non-specific lipid-transfer protein | 12.354 | 9.9741 | 24.4 | 4 | Up | 7.7332E-07 |
| AUR62006386-RA | Non-specific lipid-transfer protein | 29.514 | -2 | 14.2 | 4 | Up | 2.9296E-06 |
| AUR62020909-RA | Oil body-associated protein 1A | 22.683 | 29.641 | 53.2 | 7 | Up | 1.9218E-07 |
| AUR62018510-RA | Oil body-associated protein 1A | 19.332 | 73.376 | 74.9 | 10 | Up | 0.00127751 |
| AUR62044500-RA | Oil body-associated protein 2A | 26.809 | 55.821 | 67.5 | 14 | Up | 2.7993E-08 |
| AUR62036611-RA | Oil body-associated protein 2A | 26.883 | 139.92 | 67.5 | 14 | Up | 4.6465E-07 |
| AUR62006110-RA | Probable plastid-lipid-associated protein 14, chloroplastic isoform X1 | 74.644 | 2.1617 | 1.4 | 1 | Up | 0.000003554 |
| AUR62028367-RA | Probable peroxygenase 4 | 22.114 | 9.7066 | 23.9 | 4 | Up | 1.4974E-06 |
| AUR62031162-RA | Phospholipase A1-IIgamma | 24.759 | 5.0743 | 4.1 | 1 | Up | 0.00033922 |
| AUR62013392-RA | Glycerophosphodiester phosphodiesterase GDPD4 isoform X1 | 33.987 | 1.1508 | 3 | 1 | Up | 0.009642 |
| AUR62036552-RA | GDSL esterase/lipase At5g45920 | 26.339 | 2.7328 | 6.8 | 2 | Up | 0.00040482 |
| **Amino acid metabolism** | |  |  |  |  |  |  |
| AUR62006803-RA | S-adenosylmethionine synthase 1 | 30.154 | 100.83 | 53.6 | 10 | Down | 3.4602E-06 |
| AUR62011730-RA | S-adenosylmethionine synthase 1 | 29.957 | 25.533 | 45.4 | 8 | Down | 3.1142E-06 |
| AUR62024299-RA | Tryptophan aminotransferase-related protein 1 | 39.852 | 1.2364 | 2 | 1 | Down | 0.00165509 |
| AUR62011950-RA | Tryptophan synthase alpha chain isoform X1 | 35.863 | 1.6069 | 23.1 | 5 | Down | 0.00155771 |
| AUR62028658-RA | Serine carboxypeptidase-like 20 | 56.035 | 23.925 | 8.2 | 3 | Down | 0.04252 |
| AUR62030292-RA | Serine carboxypeptidase-like 45 | 53.408 | 81.918 | 31.5 | 12 | Down | 0.000139958 |
| AUR62009085-RA | Probable S-adenosylmethionine-dependent methyltransferase | 40.84 | 11.397 | 13.1 | 4 | Down | 0.030823 |
| AUR62017664-RA | Delta(24)-sterol reductase | 65.383 | 69.041 | 27.4 | 14 | Down | 0.00006049 |
| AUR62010123-RA | Glutamate decarboxylase | 48.13 | 4.9689 | 20.3 | 8 | Down | 2.6652E-07 |
| AUR62009082-RA | Probable S-adenosylmethionine-dependent methyltransferase | 39.765 | 13.751 | 23.4 | 7 | Up | 0.000020342 |
| **Protein related to nutrition storage** | |  |  |  |  |  |  |
| AUR62019367-RA | Bark storage protein A | 35.871 | 19.853 | 15.9 | 5 | Down | 1.57516E-05 |
| AUR62006123-RA | Bark storage protein A | 35.052 | 101.77 | 41.8 | 11 | Down | 0.000037219 |
| AUR62019363-RA | Bark storage protein A | 35.781 | 21.707 | 28.5 | 8 | Down | 1.6695E-07 |
| AUR62006122-RA | Bark storage protein A | 32.63 | 4.9812 | 7.7 | 2 | Down | 0.0095562 |
| AUR62043514-RA | Vacuolar-processing enzyme-like | 64.614 | 10.388 | 5.9 | 4 | Down | 0.000142012 |
| AUR62036450-RA | BURP domain protein USPL1 | 51.934 | 3.9207 | 6.8 | 3 | Down | 0.0025989 |
| AUR62017095-RA | BURP domain protein USPL1 | 62.313 | 16.399 | 16.7 | 7 | Down | 0.000037054 |
| AUR62043729-RA | BURP domain-containing protein BNM2A | 46.347 | 52.241 | 24.6 | 11 | Down | 0.000056483 |
| AUR62017893-RA | BURP domain-containing protein BNM2A | 49.516 | 5.4831 | 21 | 11 | Down | 0.00091579 |
| AUR62036943-RA | Oleosin 1 | 11.912 | 4.2961 | 16.5 | 2 | Up | 0.00083961 |
| AUR62040213-RA | Oleosin 1 | 18.272 | 10.159 | 28.7 | 5 | Up | 0.00007635 |
| AUR62008167-RA | Oleosin 18.2 kDa | 18.195 | 21.123 | 17.8 | 3 | Up | 8.6854E-07 |
| AUR62012221-RA | Oleosin 18.2 kDa | 19.03 | 39.98 | 30.4 | 8 | Up | 1.84482E-05 |
| AUR62024716-RA | 11S globulin seed storage protein 2 isoform X2 | 52.44 | 323.31 | 46.2 | 22 | Up | 0.00065615 |
| AUR62002139-RA | 13S globulin seed storage protein 1 | 109.47 | 323.31 | 47 | 36 | Up | 2.3414E-07 |
| AUR62015569-RA | 13S globulin seed storage protein 1 | 51.986 | 119.18 | 28.8 | 10 | Up | 1.63193E-05 |
| AUR62020540-RA | 2S seed storage protein | 15.358 | 1.2504 | 17.8 | 3 | Up | 0.00071733 |
| AUR62015663-RA | 2S seed storage protein | 15.553 | 3.6808 | 17.7 | 3 | Up | 0.000041216 |
| AUR62022079-RA | Vignain | 40.419 | 1.5088 | 20.8 | 8 | Up | 0.0009354 |
| AUR62024218-RA | Vignain | 39.153 | 18.416 | 37.6 | 11 | Up | 1.0246E-06 |
| AUR62005521-RA | Vignain | 39.296 | 81.48 | 39.5 | 12 | Up | 0.037943 |
| AUR62003182-RA | Vicilin-like seed storage protein At2g18540 | 101.38 | 62.939 | 15.1 | 15 | Up | 3.6558E-08 |
| AUR62025011-RA | Vicilin-like seed storage protein At2g28490 isoform X2 | 52.096 | 38.086 | 36.5 | 15 | Up | 0.0030963 |
| AUR62006523-RA | Vicilin-like seed storage protein At2g28490 isoform X2 | 58.202 | 107.75 | 32.1 | 16 | Up | 4.7523E-07 |
| AUR62034727-RA | Vicilin-like antimicrobial peptides 2-2 | 56.232 | 58.784 | 25.6 | 14 | Up | 0.0084164 |
| AUR62016063-RA | Vicilin-like antimicrobial peptides 2-2 | 52.39 | 12.418 | 11.8 | 5 | Up | 1.2739E-06 |
| AUR62033661-RA | Vicilin-like antimicrobial peptides 2-2 | 63.665 | 45.722 | 24.1 | 14 | Up | 0.00048252 |
| AUR62028591-RA | Vicilin-like antimicrobial peptides 2-3 | 47.63 | 33.361 | 31 | 12 | Up | 0.000061563 |
| AUR62032318-RA | Vicilin-like antimicrobial peptides 2-3 | 47.493 | 77.514 | 32 | 13 | Up | 4.6635E-06 |
| AUR62024712-RA | Legumin A | 51.26 | 54.834 | 47.9 | 19 | Up | 1.0548E-07 |
| AUR62011869-RA | Legumin A | 53.576 | 193.91 | 46.3 | 19 | Up | 0.00021846 |
| AUR62021514-RA | Basic 7S globulin | 47.013 | 25.873 | 24.4 | 9 | Up | 3.9045E-07 |
| **mRNA transcription** | |  |  |  |  |  |  |
| AUR62020042-RA | Protein plastid transcriptionally active 16, chloroplastic | 54.448 | 69.674 | 30.4 | 13 | Down | 1.6705E-06 |
| AUR62034384-RA | Transcription termination factor MTERF4, chloroplastic | 52.056 | 1.2514 | 3.5 | 2 | Down | 0.000081059 |
| AUR62032222-RA | Transcription factor bHLH3 | 16.068 | 22.049 | 22.1 | 3 | Down | 0.00157658 |
| AUR62028687-RA | Factor of DNA methylation 1 | 76.221 | 4.171 | 10.2 | 6 | Down | 0.00017861 |
| AUR62008109-RA | Factor of DNA methylation 1 | 76.606 | 15.791 | 12.5 | 8 | Down | 0.000040951 |
| AUR62028152-RA | DNA (cytosine-5)-methyltransferase CMT2 | 87.181 | 5.7154 | 4.7 | 3 | Down | 0.019616 |
| AUR62005079-RA | Increased DNA methylation 2 | 42.288 | 5.7388 | 5.9 | 2 | Down | 0.000115278 |
| AUR62028844-RA | Trihelix transcription factor ASR3-like isoform X2 | 26.786 | 1.6749 | 7.8 | 1 | Down | 0.00124311 |
| AUR62018599-RA | Probable methyltransferase PMT21 | 67.156 | 3.9373 | 9.7 | 5 | Down | 0.000101538 |
| AUR62007683-RA | Probable methyltransferase PMT26 | 84.753 | 53.045 | 12.9 | 10 | Down | 0.000137278 |
| AUR62013470-RA | Petal death protein | 32.69 | 5.8782 | 27.4 | 7 | Down | 0.00067618 |
| AUR62026801-RA | Petal death protein | 32.829 | 10.041 | 25.7 | 8 | Down | 0.000019515 |
| AUR62004217-RA | AT-hook motif nuclear-localized protein 7-like | 35.636 | 3.4888 | 9.1 | 2 | Down | 0.00092278 |
| AUR62036774-RA | Methyl-CpG-binding domain-containing protein 4-like | 20.668 | 0.95924 | 5.6 | 1 | Down | 4.2988E-06 |
| AUR62012498-RA | Lysine-specific demethylase 3B | 104.32 | 1.44 | 1.7 | 1 | Down | 0.0073633 |
| AUR62007141-RA | Histone-lysine N-methyltransferase, H3 lysine-9 specific SUVH6 | 49.604 | 0.99867 | 2.7 | 1 | Up | 0.00002466 |
| AUR62042086-RA | Transcription factor DIVARICATA | 27.509 | 4.4814 | 11.1 | 3 | Up | 0.000075774 |
| AUR62027079-RA | Transcription factor GTE7 | 61.998 | 3.4501 | 3.4 | 2 | Up | 3.1113E-06 |
| AUR62019579-RA | Multiprotein-bridging factor 1a-like isoform X2 | 15.47 | 8.2223 | 23.9 | 3 | Up | 0.00138475 |
| AUR62013337-RA | Methyl-CpG-binding domain-containing protein 11 | 47.683 | 17.892 | 8.9 | 3 | Up | 0.0048021 |
| AUR62014478-RA | DNA polymerase epsilon catalytic subunit A | 16.463 | 17.15 | 16.8 | 3 | Up | 3.7521E-07 |
| AUR62043895-RA | DNA topoisomerase 1 | 123.82 | 3.2144 | 2.6 | 3 | Up | 0.00047845 |
| AUR62025496-RA | GATA transcription factor-like protein | 22.936 | 9.7178 | 14.1 | 2 | Up | 0.000102325 |
| AUR62040470-RA | RNA-binding protein CP29B, chloroplastic | 21.605 | 26.351 | 38 | 7 | Up | 0.000039706 |
| AUR62013759-RA | RNA-binding protein FUS | 37.077 | 3.7969 | 22.1 | 5 | Up | 0.00094158 |
| AUR62014644-RA | DAG protein, chloroplastic | 26.474 | 2.5865 | 6.8 | 2 | Up | 1.6399E-07 |
| AUR62003961-RA | Upstream activation factor subunit UAF30 | 14.886 | 4.7473 | 17.3 | 2 | Up | 0.0026815 |
| AUR62039322-RA | Polyadenylate-binding protein RBP47 | 71.679 | 10.716 | 6.1 | 4 | Up | 0.0087581 |
| AUR62020959-RA | Nucleolin | 36.204 | 2.3283 | 6.5 | 2 | Up | 0.0022405 |
| AUR62001800-RA | H/ACA ribonucleoprotein complex subunit 4 | 60.521 | 12.514 | 8.5 | 4 | Up | 0.0050751 |
| AUR62007597-RA | GTPase Era | 37.765 | 4.8277 | 9.3 | 3 | Up | 0.000156922 |
| **Protein metabolism** | |  |  |  |  |  |  |
| AUR62025890-RA | Ribosomal protein S7 (chloroplast) | 17.356 | 9.0564 | 12.3 | 2 | Up | 1.74955E-05 |
| AUR62004702-RA | 30S ribosomal protein S13, chloroplastic | 19.418 | 9.2059 | 16.2 | 3 | Up | 0.000044728 |
| AUR62011660-RA | 30S ribosomal protein S17, chloroplastic | 18.525 | 9.4273 | 26.6 | 6 | Up | 3.5519E-07 |
| AUR62036161-RA | 30S ribosomal protein S20, chloroplastic | 19.969 | 6.5504 | 15.3 | 2 | Up | 1.90076E-05 |
| AUR62040078-RA | 30S ribosomal protein S9, chloroplastic | 20.072 | 15.215 | 7.5 | 1 | Up | 0.00057949 |
| AUR62008996-RA | 50S ribosomal protein HLP, mitochondrial isoform X1 | 18.173 | 1.4716 | 5.9 | 1 | Up | 0.000038995 |
| AUR62036014-RA | 50S ribosomal protein L10, chloroplastic | 25.793 | 36.688 | 40.8 | 8 | Up | 0.000140018 |
| AUR62019642-RA | 50S ribosomal protein L13, chloroplastic | 27.688 | 6.2812 | 13.4 | 3 | Up | 0.0055553 |
| AUR62002860-RA | 50S ribosomal protein L15, chloroplastic | 34.689 | 18.851 | 20 | 6 | Up | 0.00035809 |
| AUR62035212-RA | 50S ribosomal protein L15, chloroplastic | 22.025 | 6.3588 | 30.7 | 6 | Up | 0.00004298 |
| AUR62008494-RA | 50S ribosomal protein L17, chloroplastic-like | 23.508 | 1.6715 | 7.5 | 2 | Up | 0.000020501 |
| AUR62023677-RA | 50S ribosomal protein L18, chloroplastic | 18.366 | 5.5219 | 17 | 4 | Up | 0.00004494 |
| AUR62004644-RA | 50S ribosomal protein L21, chloroplastic | 25.688 | 3.5608 | 18.6 | 5 | Up | 0.00111655 |
| AUR62017613-RA | 50S ribosomal protein L23, chloroplastic | 26.047 | 7.6677 | 11.8 | 3 | Up | 0.000056448 |
| AUR62038001-RA | 50S ribosomal protein L3, chloroplastic | 30.366 | 35.739 | 24.9 | 6 | Up | 1.64077E-05 |
| AUR62001682-RA | 50S ribosomal protein L4, chloroplastic | 33.306 | 35.763 | 22.3 | 6 | Up | 0.000018486 |
| AUR62025126-RA | 50S ribosomal protein L6, chloroplastic | 23.6 | 15.828 | 26.9 | 5 | Up | 0.00121729 |
| AUR62014977-RA | 50S ribosomal protein L9, chloroplastic | 22.042 | 34.83 | 28.6 | 7 | Up | 0.00040086 |
| AUR62028474-RA | 40S ribosomal protein S13 | 17.111 | 11.718 | 29.8 | 4 | Up | 0.00051539 |
| AUR62024465-RA | 40S ribosomal protein S16 | 16.905 | 33.365 | 41.2 | 8 | Up | 3.8333E-07 |
| AUR62024433-RA | 40S ribosomal protein S2-2 | 29.878 | 56.069 | 39.3 | 12 | Up | 2.7977E-08 |
| AUR62027912-RA | 40S ribosomal protein S23 | 15.697 | 10.975 | 14.8 | 4 | Up | 3.6815E-06 |
| AUR62023978-RA | 40S ribosomal protein S24-1 | 18.243 | 26.531 | 30.2 | 5 | Up | 0.000084731 |
| AUR62016761-RA | 40S ribosomal protein S25 | 12.42 | 19.4 | 30.7 | 4 | Up | 0.000018637 |
| AUR62040675-RA | 40S ribosomal protein S8 isoform X2 | 24.604 | 1.7443 | 38.9 | 7 | Up | 0.02904 |
| AUR62006520-RA | 60S ribosomal protein L13a-2 | 22.761 | 20.199 | 25.3 | 6 | Up | 0.00021525 |
| AUR62015551-RA | 60S ribosomal protein L14-1 | 15.183 | 1.2209 | 39.1 | 5 | Up | 0.00136401 |
| AUR62037594-RA | 60S ribosomal protein L14-1 | 15.222 | 19.847 | 39.1 | 5 | Up | 0.00136208 |
| AUR62025925-RA | 60S ribosomal protein L17-2 | 19.56 | 35.7 | 32.4 | 7 | Up | 1.4664E-06 |
| AUR62025559-RA | 60S ribosomal protein L18-2 | 20.828 | 20.98 | 29.9 | 5 | Up | 0.00107564 |
| AUR62030215-RA | 60S ribosomal protein L18a-1 | 21.326 | 9.341 | 16.9 | 4 | Up | 0.00082032 |
| AUR62035092-RA | 60S ribosomal protein L23 | 13.422 | 27.22 | 64.8 | 6 | Up | 1.50768E-05 |
| AUR62020844-RA | 60S ribosomal protein L23a | 17.413 | 5.2847 | 8.4 | 1 | Up | 0.000042277 |
| AUR62040244-RA | 60S ribosomal protein L26-1 | 16.624 | 5.0558 | 19.2 | 3 | Up | 2.2682E-07 |
| AUR62038339-RA | 60S ribosomal protein L28-1 | 16.693 | 9.3852 | 38.9 | 6 | Up | 0.00029651 |
| AUR62023907-RA | 60S ribosomal protein L28-1 | 16.676 | 3.5838 | 38.9 | 6 | Up | 0.000178693 |
| AUR62020167-RA | 60S ribosomal protein L30 | 12.375 | 43.171 | 32.4 | 4 | Up | 0.0000151 |
| AUR62036284-RA | 60S ribosomal protein L32-1-like | 15.569 | 16.646 | 32.3 | 3 | Up | 0.00060377 |
| AUR62023381-RA | 60S ribosomal protein L37a isoform X2 | 10.255 | 4.591 | 26.1 | 2 | Up | 0.00036143 |
| AUR62006630-RA | 60S ribosomal protein L38 | 8.0385 | 7.5916 | 53.6 | 3 | Up | 0.0030621 |
| AUR62030870-RA | 60S ribosomal protein L8-1 | 28.254 | 52.3 | 36.5 | 8 | Up | 0.000118647 |
| AUR62006005-RA | Ribosome-binding factor PSRP1, chloroplastic | 32.268 | 13.82 | 17.1 | 6 | Up | 0.000104722 |
| AUR62035622-RA | Ribosome-binding factor PSRP1, chloroplastic | 33.964 | 14.985 | 16.2 | 6 | Up | 0.000055715 |
| AUR62042273-RA | UDP-glycosyltransferase 73C2 | 38.85 | 2.2114 | 4.3 | 2 | Up | 0.000157605 |
| AUR62008660-RA | AAA-type ATPase family protein | 135.15 | 38.821 | 5.3 | 5 | Down | 0.00149736 |
| AUR62006432-RA | Serine/threonine-protein kinase STN8, chloroplastic | 55.008 | 3.4463 | 3.4 | 2 | Down | 0.00020443 |
| AUR62007563-RA | Random slug protein 5 | 31.046 | 1.2882 | 4.1 | 1 | Down | 4.3948E-06 |
| AUR62004048-RA | Random slug protein 5 | 34.892 | 1.0054 | 3.6 | 1 | Down | 0.0031831 |
| AUR62013647-RA | Protein ASPARTIC PROTEASE IN GUARD CELL 1 | 38.025 | 73.051 | 17.2 | 6 | Down | 0.0033177 |
| AUR62014609-RA | Putative receptor-like protein kinase At4g00960 | 44.82 | 1.0845 | 2.5 | 1 | Down | 0.00021985 |
| AUR62025641-RA | GDP-fucose protein O-fucosyltransferase | 23.656 | 20.234 | 10.8 | 2 | Down | 0.000076647 |
| AUR62036451-RA | Glutaminyl-peptide cyclotransferase isoform X1 | 30.668 | 5.1508 | 7.2 | 2 | Down | 0.000039974 |
| AUR62021285-RA | Subtilisin-like protease SBT1.3 | 78.662 | 7.8058 | 4.6 | 3 | Down | 0.000060247 |
| AUR62009909-RA | Subtilisin-like protease SBT1.4 | 82.131 | 22.041 | 6.2 | 4 | Down | 0.000183992 |
| AUR62001460-RA | Subtilisin-like protease SBT1.4 | 78.725 | 4.6914 | 2.7 | 2 | Down | 0.000036503 |
| AUR62025523-RA | Subtilisin-like protease SBT1.5 | 81.378 | 12.486 | 18.5 | 9 | Down | 0.000103328 |
| AUR62008712-RA | Subtilisin-like protease SBT1.5 | 81.553 | 131.43 | 22.9 | 16 | Down | 0.008045 |
| AUR62038784-RA | Subtilisin-like protease SBT1.7 | 77.435 | 1.0151 | 3.1 | 2 | Down | 0.000018761 |
| AUR62010510-RA | Putative transferase At4g12130, mitochondrial | 42.332 | 28.792 | 18.5 | 7 | Down | 6.979E-08 |
| AUR62017391-RA | Putative transferase At4g12130, mitochondrial | 26.093 | 3.9735 | 20.4 | 4 | Down | 0.000042583 |
| AUR62019453-RA | Probable protein phosphatase 2C 39 isoform X2 | 31.562 | 52.308 | 19.9 | 5 | Down | 2.8915E-06 |
| AUR62036176-RA | Probable protein phosphatase 2C 39 isoform X3 | 31.782 | 14.454 | 14.6 | 4 | Down | 1.54452E-05 |
| AUR62022089-RA | Protein-L-isoaspartate O-methyltransferase isoform X1 | 24.921 | 20.914 | 33.8 | 6 | Down | 0.0023223 |
| AUR62019206-RA | LOW QUALITY PROTEIN: casein kinase 1-like protein HD16 | 72.473 | 1.8169 | 2.3 | 2 | Down | 0.0039213 |
| AUR62019473-RA | 26S proteasome non-ATPase regulatory subunit | 50.737 | 61.163 | 24.9 | 9 | Down | 6.5124E-08 |
| AUR62039949-RA | 36.4 kDa proline-rich protein | 25.347 | 1.1786 | 3.2 | 1 | Down | 0.00042489 |
| AUR62001894-RA | Gamma-interferon-inducible lysosomal thiol reductase | 46.163 | 2.4747 | 4.3 | 2 | Down | 0.000018266 |
| AUR62042842-RA | Eukaryotic peptide chain release factor subunit 1-3 | 56.709 | -2 | 10.5 | 5 | Down | 0.00095867 |
| **Protein related to transportation** | |  |  |  |  |  |  |
| AUR62042509-RA | ABC transporter B family member 1 | 147.49 | 15.564 | 2.7 | 3 | Down | 0.002999 |
| AUR62018692-RA | ABC transporter B family member 2-like | 131.58 | 1.1607 | 3 | 3 | Down | 0.00174209 |
| AUR62044481-RA | Miraculin | 23.288 | 23.316 | 14.2 | 3 | Down | 0.00145725 |
| AUR62044108-RA | Miraculin | 32.265 | 32.826 | 12.4 | 4 | Down | 0.00032427 |
| AUR62017240-RA | Tubulin beta-1 chain | 50.023 | 44.076 | 41.2 | 13 | Down | 0.00108241 |
| AUR62000222-RA | Tubulin beta-4 chain | 50.181 | 102.47 | 41 | 13 | Down | 0.0052155 |
| AUR62006568-RA | Tubulin beta-4 chain | 50.193 | 4.9661 | 41 | 13 | Down | 0.000081084 |
| AUR62015882-RA | 65-kDa microtubule-associated protein 1 | 57.219 | 63.753 | 27.5 | 13 | Down | 0.00141767 |
| AUR62020341-RA | 65-kDa microtubule-associated protein 1 | 57.214 | 18.743 | 24.2 | 11 | Down | 0.000115518 |
| AUR62012393-RA | Protein WVD2-like 6 isoform X1 | 52.91 | 1.5403 | 2.1 | 1 | Down | 0.0050374 |
| AUR62029724-RA | Non-specific lipid-transfer protein | 25.757 | 40.374 | 30.2 | 8 | Down | 0.00139627 |
| AUR62039110-RA | Transmembrane 9 superfamily member 2 | 68.225 | 17.574 | 13.8 | 9 | Down | 0.000084936 |
| AUR62001138-RA | Ras GTPase-activating protein-binding protein 1 | 46.418 | 6.5142 | 9 | 4 | Down | 0.0033988 |
| AUR62041757-RA | Short-chain dehydrogenase TIC 32, chloroplastic | 30.839 | 2.1744 | 3.2 | 1 | Down | 0.00185565 |
| AUR62014057-RA | ADP-ribosylation factor 2 | 20.708 | 2.0629 | 38.7 | 6 | Down | 0.015882 |
| AUR62000545-RA | Protein disulfide isomerase-like 1-1 | 58.595 | 2.952 | 23.7 | 11 | Down | 0.00101786 |
| AUR62039203-RA | Proton pump-interactor 1-like | 68.629 | 52.648 | 30.2 | 19 | Down | 0.000101514 |
| AUR62020606-RA | Plasma membrane ATPase 4 isoform X1 | 105.03 | 39.428 | 18.7 | 17 | Down | 0.0033374 |
| AUR62037160-RA | Protein argonaute 4 | 107.63 | 99.317 | 24.6 | 21 | Down | 6.5763E-07 |
| AUR62022452-RA | Probable sugar phosphate/phosphate translocator At3g17430 isoform X2 | 34.986 | 0.98943 | 2.8 | 1 | Down | 0.0034005 |
| AUR62016134-RA | GTP-binding protein SAR1A | 48.801 | 2.8087 | 23.7 | 7 | Down | 0.0167611 |
| AUR62002740-RA | Katanin p60 ATPase-containing subunit A-like 2 | 43.059 | 4.7782 | 9.4 | 4 | Down | 0.00012027 |
| AUR62039857-RA | Glucan endo-1,3-beta-glucosidase-like precursor | 36.554 | 43.984 | 23.5 | 6 | Up | 0.000039955 |
| AUR62016131-RA | Outer envelope pore protein 16-2, chloroplastic | 19.64 | 21.958 | 21 | 4 | Up | 0.0017417 |
| AUR62006721-RA | Transmembrane protein, putative | 94.484 | 8.8046 | 5.1 | 4 | Up | 1.62113E-05 |
| AUR62036972-RA | Transmembrane protein, putative | 15.363 | 35.646 | 41.3 | 5 | Up | 0.00037645 |
| AUR62032414-RA | Transmembrane protein | 10.125 | 8.732 | 39.4 | 4 | Up | 0.00028236 |
| AUR62024806-RA | Transmembrane protein | 13.315 | 19.952 | 39 | 5 | Up | 1.54867E-05 |
| AUR62004311-RA | Transmembrane protein | 8.9693 | 7.6413 | 43.2 | 3 | Up | 0.00056111 |
| AUR62022130-RA | High-affinity nitrate transporter 3.2 | 21.83 | 1.1561 | 4 | 1 | Up | 1.67684E-05 |
| AUR62006782-RA | WAT1-related protein At1g09380 | 39.06 | 1.3141 | 2.2 | 1 | Up | 0.00020368 |
| AUR62005718-RA | Protein NRT1/ PTR FAMILY 6.1 | 68.782 | 1.189 | 2.4 | 2 | Up | 1.61271E-05 |
| AUR62034626-RA | Protein NETWORKED 3A | 55.378 | 1.7429 | 2.1 | 1 | Up | 1.1229E-06 |
| AUR62012951-RA | Pentatricopeptide repeat-containing protein At3g29230 | 154.83 | 33.101 | 9 | 11 | Up | 0.00096181 |
| AUR62035579-RA | Oligopeptide transporter 7 | 69.288 | 1.2613 | 1.1 | 1 | Up | 0.00019675 |
| **Phytohormone biosynthesis and signal transduction** | |  |  |  |  |  |  |
| AUR62013932-RA | 1-aminocyclopropane-1-carboxylate oxidase homolog 1-like | 27.218 | 2.3194 | 8.8 | 2 | Down | 0.00009607 |
| AUR62002064-RA | 4-coumarate--CoA ligase 2 | 62.524 | 20.049 | 6.3 | 3 | Down | 0.000057857 |
| AUR62009768-RA | Alpha/beta fold hydrolase | 28.73 | 7.3406 | 7.5 | 2 | Down | 0.000023638 |
| AUR62009767-RA | Alpha/beta fold hydrolase | 12.088 | 6.6437 | 21.9 | 2 | Down | 0.000020928 |
| AUR62013246-RA | Leucine-rich repeat (LRR) family protein | 38.055 | 11.757 | 22.6 | 9 | Down | 2.8916E-06 |
| AUR62040875-RA | DCN1-like protein 2 isoform X2 | 23.43 | 1.9123 | 3.9 | 1 | Down | 0.0068181 |
| AUR62029574-RA | 11-beta-hydroxysteroid dehydrogenase 1B isoform X1 | 38.666 | 6.8037 | 14.6 | 5 | Up | 0.011363 |
| AUR62038844-RA | 11-beta-hydroxysteroid dehydrogenase 1B-like | 22.158 | 36.68 | 54.8 | 10 | Up | 3.297E-07 |
| AUR62029572-RA | 11-beta-hydroxysteroid dehydrogenase 1B-like | 37.136 | 60.658 | 44.3 | 13 | Up | 1.6331E-07 |
| AUR62038845-RA | 11-beta-hydroxysteroid dehydrogenase 1B-like | 69.791 | 7.3223 | 11.3 | 7 | Up | 0.000057805 |
| AUR62022399-RA | 11-beta-hydroxysteroid dehydrogenase-like 5 | 39.507 | 15.364 | 12.5 | 4 | Up | 1.61473E-05 |
| AUR62024495-RA | Zeatin O-glucosyltransferase | 53.106 | 7.4374 | 2.8 | 1 | Up | 0.000037645 |
| AUR62034234-RA | LanC-like protein GCR2 | 45.124 | 1.4894 | 5.4 | 2 | Up | 0.00021613 |
| AUR62035880-RA | AWPM-19-like family protein | 19.037 | 13.087 | 13.6 | 2 | Up | 0.000024112 |
| **Cell development** | |  |  |  |  |  |  |
| AUR62011272-RA | DNA replication licensing factor MCM5 | 74.121 | 21.356 | 5.4 | 3 | Down | 0.00022198 |
| AUR62024658-RA | DNA replication licensing factor MCM7 | 78.548 | 7.3454 | 4.2 | 3 | Down | 0.037502 |
| AUR62039793-RA | Structural maintenance of chromosomes protein 4 | 129.58 | 4.1282 | 2.2 | 3 | Down | 0.000044432 |
| AUR62043832-RA | Protein POLLENLESS 3-LIKE 2 | 40.844 | 1.6069 | 2.5 | 1 | Down | 0.00021697 |
| AUR62027754-RA | Synaptonemal complex protein 1-like isoform X2 | 93.449 | 0.99222 | 0.9 | 1 | Down | 0.00057736 |
| AUR62016318-RA | Protein TSS | 162.98 | 3.4391 | 1.8 | 3 | Down | 0.000023283 |
| AUR62021255-RA | Protein HOTHEAD | 61.858 | 1.8968 | 3 | 2 | Down | 0.000083756 |
| AUR62001255-RA | AT-rich interactive domain-containing protein 3-like | 69.455 | 2.2172 | 6.4 | 3 | Down | 0.00041939 |
| AUR62021123-RA | Probable polygalacturonase | 52.752 | 3.4989 | 11.8 | 6 | Down | 0.00103954 |
| AUR62031421-RA | Probable polygalacturonase | 100.73 | 33.236 | 6.1 | 6 | Down | 0.000057977 |
| AUR62003605-RA | Probable pectinesterase/pectinesterase inhibitor 51 | 29.542 | 4.0502 | 9 | 3 | Down | 0.00079971 |
| AUR62007974-RA | Probable xyloglucan endotransglucosylase/hydrolase protein 6 | 33.488 | 10.033 | 13.8 | 3 | Down | 0.00020313 |
| AUR62001254-RA | Probable pectate lyase 18 | 44.737 | 3.6585 | 7.7 | 2 | Down | 0.000001767 |
| AUR62003682-RA | Probable glycosyltransferase At5g03795 | 38.193 | 16.822 | 5.8 | 2 | Down | 0.00050271 |
| AUR62032292-RA | Caffeoylshikimate esterase | 32.694 | 11.526 | 12.2 | 3 | Down | 0.00006363 |
| AUR62026804-RA | Basic blue protein | 13.391 | 2.9976 | 15.9 | 2 | Down | 0.00044484 |
| AUR62020703-RA | Apyrase 2 | 50.678 | 4.2235 | 3.9 | 2 | Down | 1.2366E-06 |
| AUR62001903-RA | Filament-like plant protein 7 | 102.79 | 1.1027 | 1 | 1 | Down | 0.0120032 |
| AUR62013778-RA | Glycine-rich cell wall structural protein 1.8 | 21.69 | 74.779 | 37 | 4 | Up | 0.000082174 |
| AUR62026686-RA | Glycine-rich cell wall structural protein 1.8 | 22.214 | 2.1771 | 11.6 | 2 | Up | 0.000159588 |
| AUR62037914-RA | Embryonic protein DC-8 | 52.53 | 175.9 | 54.1 | 31 | Up | 1.6223E-06 |
| AUR62040165-RA | Embryonic protein DC-8 | 53.521 | 59.709 | 53.6 | 28 | Up | 1.6657E-07 |
| AUR62014787-RA | Embryonic protein DC-8 isoform X1 | 55.544 | 96.781 | 21.7 | 13 | Up | 4.5569E-08 |
| AUR62042308-RA | Seed biotin-containing protein SBP65 | 72.07 | 120.47 | 57.1 | 35 | Up | 1.61564E-05 |
| AUR62037387-RA | Seed biotin-containing protein SBP65 | 69.608 | 241.63 | 67.1 | 38 | Up | 3.8758E-06 |
| AUR62035713-RA | Seed maturation protein PM41 | 8.6914 | 10.942 | 57.3 | 3 | Up | 0.00098415 |
| AUR62016335-RA | SNF1-related protein kinase regulatory subunit gamma-like PV42a | 41.695 | 64.121 | 29.4 | 8 | Up | 0.00002205 |
| AUR62011516-RA | SNF1-related protein kinase regulatory subunit gamma-like PV42a | 35.799 | 8.8878 | 30.3 | 8 | Up | 4.3426E-06 |
| AUR62020884-RA | Xyloglucan endotransglucosylase/hydrolase 2 | 31.761 | 13.81 | 15.8 | 3 | Up | 0.00063542 |
| AUR62010618-RA | Thaumatin-like protein 1 | 23.326 | 20.785 | 13.5 | 2 | Up | 0.0056583 |
| AUR62034669-RA | Spermatogenesis-associated protein 20 isoform X1 | 96.622 | 22.669 | 11.6 | 8 | Up | 0.000037541 |
| AUR62022695-RA | Subtilisin-like protease SBT3.1 | 10.284 | 10.444 | 52.5 | 5 | Up | 0.000121263 |
| AUR62019772-RA | Putative cell division cycle ATPase | 86.89 | 23.905 | 9.5 | 7 | Up | 6.6537E-08 |
| AUR62018948-RA | Protein EXORDIUM | 33.813 | 7.6054 | 14.6 | 3 | Up | 0.00025523 |
| AUR62027741-RA | Probable xyloglucan endotransglucosylase/hydrolase protein 23 | 31.812 | 9.922 | 11.4 | 3 | Up | 0.000056258 |
| AUR62018906-RA | Probable xyloglucan endotransglucosylase/hydrolase protein 23 | 31.923 | 6.3648 | 10.9 | 3 | Up | 1.57279E-05 |
| AUR62029826-RA | Mitochondrial fission protein ELM1 | 41.227 | 1.2045 | 2.2 | 1 | Up | 0.0045588 |
| AUR62020995-RA | Alpha-L-arabinofuranosidase 1 | 73.53 | 1.6395 | 20.5 | 10 | Up | 0.000181083 |
| AUR62018438-RA | Alpha-L-arabinofuranosidase 1 | 73.577 | 78.961 | 20.5 | 10 | Up | 0.000063639 |
| AUR62007217-RA | Em-like protein GEA6 | 9.0757 | 19.897 | 48.8 | 5 | Up | 0.000020304 |
| AUR62004613-RA | Late embryogenesis abundant protein (LEA) family protein | 50.642 | 34.847 | 24.5 | 10 | Up | 2.1907E-06 |
| AUR62022650-RA | Late embryogenesis abundant protein (LEA) family protein | 53.519 | 79.371 | 30.8 | 15 | Up | 1.57738E-05 |
| AUR62011287-RA | Late embryogenesis abundant protein | 34.231 | 76.446 | 28.7 | 7 | Up | 0.00002424 |
| AUR62007271-RA | Late embryogenesis abundant protein 18 | 14.669 | 33.916 | 35.6 | 4 | Up | 1.3421E-06 |
| AUR62018728-RA | Late embryogenesis abundant protein 18 | 14.474 | 4.0655 | 34.6 | 4 | Up | 0.000097647 |
| AUR62014840-RA | Late embryogenesis abundant protein 1-like | 30.791 | 17.503 | 37.5 | 10 | Up | 2.4044E-06 |
| AUR62032329-RA | Late embryogenesis abundant protein 31 | 26.53 | 11.005 | 21.2 | 4 | Up | 0.0036764 |
| AUR62012039-RA | Late embryogenesis abundant protein 46 | 17.938 | 25.546 | 29.2 | 5 | Up | 0.0031194 |
| AUR62032330-RA | Late embryogenesis abundant protein 47 | 15.066 | 10.012 | 31.5 | 3 | Up | 0.00101606 |
| AUR62029965-RA | Late embryogenesis abundant protein D-29 isoform X1 | 21.611 | 52.956 | 33.3 | 8 | Up | 1.8375E-06 |
| AUR62002551-RA | Late embryogenesis abundant protein D-29 isoform X1 | 26.689 | 21.176 | 30.3 | 7 | Up | 2.2202E-08 |
| AUR62028605-RA | Late embryogenesis abundant protein D-34 | 26.874 | 68.502 | 54.4 | 11 | Up | 0.000080688 |
| AUR62034707-RA | Late embryogenesis abundant protein D-34-like | 27.661 | 120.97 | 65.3 | 13 | Up | 0.000119093 |
| AUR62043549-RA | Late embryogenesis abundant protein D-34-like | 27.531 | 45.917 | 51.7 | 12 | Up | 0.00038461 |
| AUR62011567-RA | Late embryogenesis abundant protein Dc3 | 19.924 | 45.523 | 50.3 | 12 | Up | 5.8776E-09 |
| **Stress response proteins** | |  |  |  |  |  |  |
| AUR62025041-RA | L-ascorbate peroxidase, cytosolic | 27.497 | 72.371 | 50.8 | 9 | Down | 0.00062261 |
| AUR62012708-RA | Peroxidase 42 | 37.983 | 4.3657 | 7.5 | 3 | Down | 0.00029628 |
| AUR62042267-RA | Peroxidase 57 | 34.714 | 8.143 | 13.8 | 4 | Down | 0.0030751 |
| AUR62005974-RA | Peroxidase 5-like | 66.861 | 33.691 | 16.4 | 8 | Down | 0.000120743 |
| AUR62003704-RA | Peroxidase 64 | 34.106 | 4.051 | 8.5 | 3 | Down | 0.0020423 |
| AUR62004105-RA | Peroxidase 72 | 37.295 | 4.5942 | 10.8 | 3 | Down | 0.0127601 |
| AUR62007619-RA | Peroxidase 72 | 36.954 | 12.317 | 15.1 | 4 | Down | 0.00046343 |
| AUR62004107-RA | Peroxidase 72 | 36.575 | 81.807 | 46.8 | 11 | Down | 8.0617E-07 |
| AUR62018272-RA | CO(2)-response secreted protease | 81.994 | 12.436 | 5.6 | 4 | Down | 0.0001162 |
| AUR62037213-RA | Annexin D3 | 35.749 | 12.003 | 28.7 | 7 | Down | 0.0022242 |
| AUR62037212-RA | Annexin D4 | 35.559 | 4.8203 | 28.8 | 8 | Down | 0.00038223 |
| AUR62026841-RA | Annexin D4 | 35.477 | 48.134 | 28.8 | 8 | Down | 0.00058465 |
| AUR62026842-RA | Annexin-like protein RJ4 | 32.03 | 20.898 | 17.4 | 4 | Down | 0.0153033 |
| AUR62002012-RA | Annexin D2 | 34.447 | 66.746 | 53.2 | 13 | Up | 3.5665E-06 |
| AUR62003790-RA | Annexin D2 | 29.286 | 8.7144 | 51.2 | 12 | Up | 1.0669E-06 |
| AUR62019261-RA | DNA-damage-repair/toleration protein DRT100-like | 38.356 | 8.3809 | 13.9 | 5 | Down | 0.00052493 |
| AUR62023850-RA | Endochitinase | 29.494 | 76.989 | 27.7 | 5 | Down | 4.9152E-06 |
| AUR62005814-RA | Expansin-A1 | 26.15 | 1.2859 | 3.7 | 1 | Down | 0.0003581 |
| AUR62040856-RA | Jasmonate-induced protein homolog | 18.854 | 18.019 | 57.3 | 9 | Down | 0.000042156 |
| AUR62010975-RA | MLP-like protein 34 | 17.477 | 69.639 | 45.5 | 8 | Down | 0.00358 |
| AUR62025226-RA | MLP-like protein 34 | 17.653 | 15.223 | 34.2 | 7 | Down | 0.000038473 |
| AUR62021482-RA | MLP-like protein 34 | 17.634 | 20.283 | 40.3 | 8 | Down | 0.0008624 |
| AUR62005328-RA | MLP-like protein 423 | 17.131 | 82.131 | 66 | 15 | Down | 3.4613E-06 |
| AUR62006642-RA | Thioredoxin F-type, chloroplastic | 20.676 | 5.9 | 21 | 3 | Down | 0.000160213 |
| AUR62026795-RA | Thioredoxin M-type, chloroplastic | 19.942 | 32.474 | 31.1 | 6 | Down | 0.000075367 |
| AUR62026482-RA | Glutathione S-transferase U17 | 26.087 | 19.845 | 22.9 | 5 | Down | 0.000156645 |
| AUR62033162-RA | Glutathione S-transferase | 24.012 | 64.721 | 54.7 | 10 | Up | 0.00081937 |
| AUR62008609-RA | Glutathione S-transferase | 24.06 | 18.703 | 54.7 | 10 | Up | 0.000117957 |
| AUR62038872-RA | Hypersensitive-induced response protein 1 | 31.46 | 10.235 | 26.5 | 7 | Down | 0.00033692 |
| AUR62024308-RA | Kirola | 17.36 | 28.185 | 28.8 | 5 | Down | 0.000198966 |
| AUR62002313-RA | Kirola | 17.347 | 15.941 | 21.6 | 4 | Down | 0.00039728 |
| AUR62011447-RA | Natterin-1-like | 35.099 | 2.1809 | 2.3 | 1 | Down | 0.0027579 |
| AUR62024203-RA | Natterin-1-like | 59.162 | 24.841 | 21.5 | 9 | Down | 0.000082552 |
| AUR62006405-RA | Natterin-1-like | 57.16 | 42.058 | 22.2 | 8 | Down | 3.2397E-06 |
| AUR62005533-RA | Natterin-3-like | 56.505 | 7.8585 | 9 | 3 | Down | 4.5244E-06 |
| AUR62000528-RA | Ultraviolet-B receptor UVR8 | 47.848 | 42.8 | 18.6 | 8 | Down | 0.000024241 |
| AUR62023803-RA | Calcium ion-binding protein | 64.385 | 5.4348 | 5.1 | 3 | Down | 0.024282 |
| AUR62041158-RA | Mannose/glucose-specific lectin-like | 25.612 | 18.501 | 15.3 | 3 | Down | 0.0151572 |
| AUR62030976-RA | Polyamine oxidase-like | 53.349 | 129.44 | 39.7 | 19 | Down | 1.62151E-05 |
| AUR62030977-RA | Polyamine oxidase-like | 71.693 | 84.869 | 28.4 | 18 | Down | 5.3508E-07 |
| AUR62012093-RA | Probable serine/threonine-protein kinase NAK | 46.244 | 1.1208 | 4.9 | 2 | Down | 0.00113789 |
| AUR62025944-RA | Probable inactive purple acid phosphatase 27 | 69.964 | 29.694 | 17.8 | 9 | Down | 0.000076601 |
| AUR62037937-RA | Probable glutathione S-transferase parC | 26.005 | 11.195 | 19.6 | 5 | Down | 0.00183507 |
| AUR62028143-RA | Non-symbiotic hemoglobin 2 | 16.973 | 11.249 | 17.2 | 2 | Down | 1.82482E-05 |
| AUR62002040-RA | C2 domain-containing family protein | 225.05 | 12.659 | 2.9 | 6 | Down | 0.000044621 |
| AUR62021293-RA | CDGSH iron-sulfur domain-containing protein NEET | 10.966 | 5.7361 | 24.2 | 2 | Down | 0.0120594 |
| AUR62035166-RA | Cysteine protease XCP2 | 40.088 | 1.4375 | 8 | 3 | Down | 0.0026439 |
| AUR62006579-RA | Cysteine proteinase inhibitor A | 11.089 | 20.036 | 37.4 | 2 | Down | 0.0073248 |
| AUR62038240-RA | Cysteine proteinase COT44 | 40.732 | 5.0324 | 6.6 | 2 | Up | 0.000042285 |
| AUR62031684-RA | Cysteine proteinase inhibitor 6 isoform X1 | 23.541 | 8.9745 | 46.6 | 7 | Up | 0.0053184 |
| AUR62032567-RA | Cysteine proteinase inhibitor 6 isoform X1 | 23.53 | 90.58 | 46.6 | 9 | Up | 0.00109695 |
| AUR62020879-RA | Cysteine proteinase inhibitor B | 12.942 | 2.9235 | 15.5 | 2 | Up | 0.000038017 |
| AUR62024285-RA | Factor Xa inhibitor BuXI | 22.553 | 15.363 | 22.3 | 4 | Down | 0.000042919 |
| AUR62009690-RA | Gamma-interferon-inducible lysosomal thiol reductase | 28.574 | 17.36 | 17.4 | 4 | Down | 0.000042367 |
| AUR62018223-RA | 15.7 kDa heat shock protein, peroxisomal | 15.82 | 1.1571 | 13.6 | 2 | Up | 0.00196149 |
| AUR62016346-RA | 26.5 kDa heat shock protein, mitochondrial | 27.646 | 9.9524 | 11.5 | 3 | Up | 0.000035804 |
| AUR62010055-RA | Protein EARLY-RESPONSIVE TO DEHYDRATION 7, chloroplastic | 41.318 | 36.154 | 22.5 | 7 | Up | 1.73596E-05 |
| AUR62013047-RA | Protein EARLY-RESPONSIVE TO DEHYDRATION 7, chloroplastic | 38.274 | 26.771 | 24.1 | 7 | Up | 0.000040532 |
| AUR62039962-RA | Small heat shock protein, chloroplastic isoform X2 | 22.906 | 14.534 | 26.2 | 5 | Up | 0.000003652 |
| AUR62009102-RA | 17.1 kDa class II heat shock protein | 18.348 | 2.1747 | 44.5 | 7 | Up | 5.7389E-07 |
| AUR62020570-RA | 17.1 kDa class II heat shock protein | 18.276 | 57.455 | 54.3 | 8 | Up | 5.4518E-07 |
| AUR62001433-RA | 17.4 kDa class III heat shock protein | 13.241 | 15.242 | 21.7 | 2 | Up | 0.000020176 |
| AUR62012175-RA | 18.2 kDa class I heat shock protein | 18.451 | 6.7414 | 32.3 | 6 | Up | 1.87587E-05 |
| AUR62012217-RA | 18.2 kDa class I heat shock protein | 18.448 | 33.999 | 33.3 | 6 | Up | 1.61251E-05 |
| AUR62012170-RA | 18.3 kDa class I heat shock protein | 18.258 | 34.559 | 39.1 | 7 | Up | 5.0255E-07 |
| AUR62002570-RA | 1-Cys peroxiredoxin | 24.255 | 94.842 | 64.4 | 15 | Up | 0.0030387 |
| AUR62029991-RA | 1-Cys peroxiredoxin | 24.332 | 15.303 | 64.4 | 14 | Up | 0.002282 |
| AUR62017037-RA | ABA-inducible protein PHV A1 | 30.821 | 38.416 | 33.5 | 10 | Up | 1.7766E-06 |
| AUR62017046-RA | Auxin-repressed 12.5 kDa protein | 13.415 | 10.887 | 22.8 | 2 | Up | 0.000041013 |
| AUR62021118-RA | Heat shock protein 83 | 80.85 | 98.161 | 37.7 | 26 | Up | 2.4466E-06 |
| AUR62031424-RA | Heat shock protein 83 | 66.171 | 2.5577 | 39.2 | 23 | Up | 0.00076002 |
| AUR62026161-RA | Jasmonate-induced protein homolog | 18.983 | 7.6135 | 17.7 | 2 | Up | 0.00079861 |
| AUR62044335-RA | Jasmonate-induced protein homolog | 20.297 | 41.245 | 45.1 | 8 | Up | 1.72371E-05 |
| AUR62044622-RA | Jasmonate-induced protein homolog | 18.771 | 21.423 | 46.2 | 7 | Up | 1.8976E-06 |
| AUR62030818-RA | Carrot ABA-induced in somatic embryos 3 | 12.433 | 52.903 | 63.2 | 9 | Up | 0.0010215 |
| AUR62030479-RA | Carrot ABA-induced in somatic embryos 3 | 12.43 | 3.3661 | 56.1 | 8 | Up | 0.003543 |
| AUR62009027-RA | LEAF RUST 10 DISEASE-RESISTANCE LOCUS RECEPTOR-LIKE PROTEIN KINASE-like 2.4 | 54.991 | 1.0077 | 3.2 | 1 | Up | 0.0022415 |
| AUR62021191-RA | Low-temperature-induced 65 kDa protein | 84.856 | 118.05 | 27.6 | 17 | Up | 4.7636E-08 |
| AUR62021192-RA | Low-temperature-induced 65 kDa protein | 28.806 | 47.752 | 27.2 | 6 | Up | 1.83423E-05 |
| AUR62015982-RA | Low-temperature-induced 65 kDa protein | 83.92 | 6.2236 | 7.4 | 5 | Up | 0.00050398 |
| AUR62030886-RA | Pathogenesis-related protein PR-1 type | 17.677 | 1.9927 | 14.1 | 2 | Up | 1.2862E-06 |
| AUR62027039-RA | Pathogenesis-related protein PR-1 type | 17.663 | 22.431 | 19.1 | 2 | Up | 8.4581E-07 |
| AUR62030885-RA | Pathogenesis-related protein PR-1 type | 15.726 | 19.806 | 42 | 4 | Up | 0.000022209 |
| AUR62001001-RA | Pathogenesis-related protein PR-4 | 15.355 | 16.245 | 39.4 | 3 | Up | 0.00085646 |
| AUR62040443-RA | Peroxidase P7-like isoform X2 | 51.66 | 25.465 | 13.3 | 4 | Up | 0.000160332 |
| AUR62016175-RA | Peroxygenase | 28.134 | 43.161 | 39.2 | 10 | Up | 0.000139047 |
| AUR62035093-RA | Peroxygenase | 28.215 | 12.95 | 36 | 9 | Up | 4.1156E-06 |
| AUR62036292-RA | Universal stress protein A-like protein, partial | 12.61 | 5.7445 | 13.7 | 2 | Up | 0.000021309 |
| AUR62008514-RA | Chaperone protein ClpB1 isoform X1 | 100.67 | 162.91 | 28.6 | 21 | Up | 0.00028171 |
| AUR62004102-RA | Dehydrin Rab18 | 23.447 | 25.048 | 30.8 | 5 | Up | 0.000021681 |
| AUR62008645-RA | Universal stress protein PHOS34 | 14.889 | 24.394 | 27.1 | 3 | Up | 0.00138058 |
| AUR62012323-RA | Protein EARLY RESPONSIVE TO DEHYDRATION 15 | 10.221 | 2.049 | 14 | 1 | Up | 0.000096809 |
| AUR62039221-RA | Poly [ADP-ribose] polymerase 3 | 89.262 | 180.41 | 36.4 | 27 | Up | 8.4586E-07 |
| AUR62009776-RA | Poly [ADP-ribose] polymerase 3 | 90.3 | 62.575 | 40.3 | 26 | Up | 9.3022E-07 |
| AUR62027865-RA | Heme-binding-like protein At3g10130, chloroplastic | 23.101 | 30.706 | 41.4 | 8 | Up | 0.00020347 |
| AUR62019028-RA | Heme-binding-like protein At3g10130, chloroplastic | 23.158 | 2.7321 | 33.3 | 7 | Up | 0.00038135 |
| AUR62039001-RA | Probable leucine-rich repeat receptor-like protein kinase At1g35710 | 52.465 | 18.549 | 15.9 | 6 | Up | 0.000156488 |
| AUR62007657-RA | Probable leucine-rich repeat receptor-like protein kinase At1g35710 | 107.87 | 25.687 | 6.4 | 6 | Up | 0.000024959 |
| AUR62021785-RA | Transportin MOS14 | 109.32 | 3.5345 | 3.6 | 3 | Up | 0.00038397 |
| AUR62003958-RA | Peamaclein-like | 10.27 | 1.2267 | 20 | 2 | Up | 0.0030801 |
| AUR62003131-RA | Nodulin-related protein 1-like | 12.849 | 22.753 | 53.6 | 5 | Up | 0.000103974 |
| AUR62033463-RA | Early nodulin-93 | 11.404 | 17.522 | 13.9 | 1 | Up | 2.0743E-06 |
| AUR62022868-RA | Mitogen-activated protein kinase kinase kinase 12-like isoform X2 | 87.62 | 2.7811 | 2.2 | 3 | Up | 0.000077668 |
| AUR62016100-RA | Major allergen Mal d 1 | 17.729 | 47.622 | 35.4 | 4 | Up | 0.00023915 |
| AUR62016680-RA | La-related protein 1 | 75.585 | 34.013 | 9.3 | 5 | Up | 1.004E-07 |
| AUR62030943-RA | Glutaredoxin | 13.737 | 20.892 | 28.1 | 3 | Up | 2.7031E-06 |
| AUR62018748-RA | Pentatricopeptide repeat-containing protein At5g46580, chloroplastic | 80.717 | 4.709 | 7.6 | 5 | Up | 0.000022231 |
| AUR62023809-RA | Acidic endochitinase SP2 | 30.033 | 43.235 | 15.2 | 3 | Up | 0.0039368 |
| AUR62039319-RA | Calcineurin-like metallo-phosphoesterase superfamily protein | 118.46 | 1.0953 | 0.7 | 1 | Up | 0.00036409 |
| AUR62038842-RA | HVA22-like protein e | 17.388 | 1.1605 | 5.2 | 1 | Up | 1.0875E-06 |
| AUR62027785-RA | 7-deoxyloganetin glucosyltransferase | 54.229 | 1.6777 | 1.6 | 1 | Up | 0.00085982 |
| AUR62001816-RA | Dessication-induced 1VOC superfamily protein | 15.418 | 31.057 | 76.5 | 8 | Up | 1.73568E-05 |
| **Secondary metabolism** | |  |  |  |  |  |  |
| AUR62029466-RA | Naringenin,2-oxoglutarate 3-dioxygenase | 41.597 | 2.4848 | 25.7 | 8 | Down | 0.00036187 |
| AUR62028163-RA | Anthocyanidin 3-O-glucosyltransferase 7 | 92.723 | 33.889 | 10.7 | 7 | Down | 0.000079646 |
| AUR62011260-RA | Anthocyanin 3'-O-beta-glucosyltransferase-like | 55.936 | 56.269 | 24.8 | 12 | Down | 1.8034E-06 |
| AUR62022680-RA | Caffeic acid 3-O-methyltransferase | 42.136 | 23.449 | 9.4 | 4 | Down | 0.0041612 |
| AUR62009895-RA | Cytochrome P450 71A1-like | 58.781 | 5.7792 | 6.1 | 3 | Down | 0.004885 |
| AUR62005547-RA | Cytochrome P450 77A2-like | 59.262 | 8.7256 | 7.2 | 3 | Down | 0.0023375 |
| AUR62010695-RA | Cytochrome P450 86A22 | 60.001 | 1.8445 | 4 | 2 | Down | 0.000039989 |
| AUR62033991-RA | Cytochrome P450 CYP72A219-like | 65.558 | 23.79 | 5.5 | 3 | Down | 0.00056326 |
| AUR62043325-RA | Cytochrome P450 CYP82D47-like | 59.754 | 6.6925 | 7.9 | 4 | Down | 0.0070999 |
| AUR62043328-RA | Cytochrome P450 CYP82D47-like | 60.12 | 5.597 | 1.7 | 1 | Down | 1.7658E-06 |
| AUR62021145-RA | BAHD acyltransferase DCR | 51.299 | 68.803 | 26.1 | 11 | Down | 0.000075426 |
| AUR62031392-RA | BAHD acyltransferase DCR | 51.142 | 22.551 | 24.6 | 10 | Down | 0.000038421 |
| AUR62010369-RA | Benzyl alcohol O-benzoyltransferase | 50.904 | 19.294 | 12.9 | 6 | Down | 0.000042247 |
| AUR62025620-RA | 1-phosphatidylinositol-3-phosphate 5-kinase FAB1B | 23.823 | 1.7644 | 4.2 | 1 | Down | 0.00073985 |
| AUR62013199-RA | 3-hydroxy-3-methylglutaryl-coenzyme A reductase 1 | 62.311 | 8.0598 | 7.1 | 5 | Down | 0.00031977 |
| AUR62024778-RA | 3-ketoacyl-CoA synthase 10 | 61.974 | 58.884 | 20.7 | 9 | Down | 0.00023989 |
| AUR62006964-RA | 7-deoxyloganetic acid glucosyltransferase | 54.795 | 2.3964 | 15.6 | 6 | Down | 0.00025613 |
| AUR62010150-RA | 7-deoxyloganetin glucosyltransferase | 54.992 | 2.0909 | 3.1 | 2 | Down | 0.00080012 |
| AUR62042825-RA | Chalcone synthase | 43.087 | 7.3727 | 9.2 | 3 | Down | 0.000120988 |
| AUR62014351-RA | Protein ECERIFERUM 1 | 41.913 | 5.7428 | 3.5 | 1 | Down | 0.000155991 |
| AUR62041453-RA | Berberine bridge enzyme-like 10 | 60.934 | 50.926 | 20.6 | 9 | Down | 0.00039861 |
| AUR62026743-RA | Berberine bridge enzyme-like 21 | 45.509 | 4.1648 | 7 | 3 | Down | 0.00107891 |
| AUR62029565-RA | Omega-hydroxypalmitate O-feruloyl transferase | 42.864 | 2.1918 | 6.9 | 3 | Down | 2.1965E-06 |
| AUR62000272-RA | Omega-hydroxypalmitate O-feruloyl transferase | 33.901 | 1.5482 | 6.6 | 2 | Down | 0.00009901 |
| AUR62039075-RA | Probable cinnamyl alcohol dehydrogenase 1 | 38.981 | 57.608 | 33.1 | 10 | Down | 0.0046437 |
| AUR62029284-RA | (-)-germacrene D synthase-like | 127.02 | 12.513 | 8.1 | 6 | Down | 0.00041719 |
| AUR62003897-RA | Caffeoyl-CoA O-methyltransferase 2 | 28.215 | 3.9626 | 11.2 | 3 | Down | 0.000058483 |
| AUR62023632-RA | Flavonol synthase/flavanone 3-hydroxylase | 39.496 | 32.372 | 17.1 | 7 | Down | 0.000156914 |
| AUR62021178-RA | Caffeic acid 3-O-methyltransferase | 40.961 | 26.202 | 31.5 | 9 | Up | 4.4335E-06 |
| AUR62043467-RA | Putative lactoylglutathione lyase | 32.343 | 14.823 | 40.1 | 10 | Up | 0.000064119 |
| AUR62007042-RA | 2-keto-3-deoxy-L-rhamnonate aldolase | 29.213 | 3.2252 | 6.4 | 2 | Up | 0.000141712 |
| AUR62002449-RA | Cytosolic sulfotransferase 5-like | 40.143 | 4.2528 | 9.7 | 3 | Up | 0.000042458 |
| AUR62004753-RA | Cytosolic sulfotransferase 5-like | 39.661 | 9.697 | 21.3 | 6 | Up | 0.000023001 |
| **Others** | |  |  |  |  |  |  |
| AUR62043763-RA | Adenylate kinase 5, chloroplastic isoform X1 | 49.671 | 15.934 | 11.1 | 4 | Down | 1.1819E-07 |
| AUR62001683-RA | Phosphomethylpyrimidine synthase, chloroplastic isoform X2 | 72.467 | 32.025 | 7.7 | 3 | Down | 0.0054212 |
| AUR62007424-RA | Deoxyuridine 5'-triphosphate nucleotidohydrolase | 17.994 | 19.179 | 35.5 | 4 | Down | 0.00023977 |
| AUR62039186-RA | CASP-like protein 2A1 | 21.277 | 8.6979 | 11.6 | 2 | Down | 0.00047516 |
| AUR62013288-RA | Bifunctional purple acid phosphatase 26 | 146.28 | 55.591 | 7.5 | 7 | Down | 0.021475 |
| AUR62034547-RA | Purple acid phosphatase | 54.12 | 14.8 | 6.1 | 3 | Down | 0.000036554 |
| AUR62044208-RA | Phosphoethanolamine N-methyltransferase | 55.749 | 43.362 | 26.4 | 12 | Down | 0.000035134 |
| AUR62017829-RA | Ribonucleoside-diphosphate reductase small chain | 38.085 | 4.6585 | 8.1 | 3 | Down | 0.00033965 |
| AUR62038057-RA | Stem-specific protein TSJT1 | 25.376 | 34.286 | 25.4 | 4 | Down | 1.53908E-05 |
| AUR62029354-RA | Stem-specific protein TSJT1 | 28.624 | 33.131 | 24.6 | 5 | Up | 8.1213E-07 |
| AUR62016981-RA | Stem-specific protein TSJT1 | 28.21 | 14.916 | 9.7 | 3 | Up | 1.56089E-05 |
| AUR62016982-RA | Stem-specific protein TSJT1 | 27.605 | 21.204 | 13 | 4 | Up | 0.00032203 |
| AUR62026514-RA | UDP-glycosyltransferase 79B9 | 95.732 | 2.1906 | 2.5 | 3 | Down | 0.0046164 |
| AUR62004273-RA | Uracil phosphoribosyltransferase | 29.274 | 31.578 | 34.5 | 8 | Down | 0.00125721 |
| AUR62005583-RA | Urease | 86.16 | 2.3524 | 2.7 | 2 | Up | 0.049256 |
| AUR62007083-RA | Soluble inorganic pyrophosphatase 1 isoform X2 | 25.347 | -2 | 15.8 | 3 | Up | 0.00028199 |
| AUR62005794-RA | Ribonuclease 1 | 26.812 | 11.61 | 19 | 5 | Up | 2.8664E-06 |
| AUR62003629-RA | Putative nuclease HARBI1 | 32.766 | 4.0009 | 11.7 | 3 | Up | 0.000039123 |
| AUR62012905-RA | Phage capsid scaffolding protein (GPO) serine peptidase | 23.943 | 7.6401 | 21.2 | 4 | Up | 0.000198644 |
| AUR62036969-RA | Glutamyl-tRNA(Gln) amidotransferase subunit A isoform X1 | 70.779 | 19.575 | 12.7 | 7 | Up | 3.8293E-06 |
| **Unknown proteins** | |  |  |  |  |  |  |
| AUR62044161-RA | ArgH (DUF639) | 70.964 | 12.859 | 9.1 | 4 | Up | 0.00021851 |
| AUR62020116-RA | Pleckstrin homology domain-containing protein 1 | 16.546 | 1.7544 | 5.6 | 1 | Up | 0.0029002 |
| AUR62005135-RA | Uncharacterized GPI-anchored protein At3g06035 | 20.88 | 11.031 | 26 | 3 | Up | 0.00005844 |
| AUR62034376-RA | Uncharacterized oxidoreductase At4g09670 isoform X3 | 40.623 | 23.807 | 17.6 | 5 | Up | 0.000102351 |
| AUR62006769-RA | Plant/protein (Protein of unknown function, DUF538) | 21.752 | 28.257 | 23.4 | 3 | Down | 0.000157606 |
| AUR62002461-RA | DPP6 N-terminal domain-like protein | 61.895 | 1.1817 | 19.3 | 9 | Down | 0.00130044 |
| AUR62014822-RA | DUF2921 family protein | 92.15 | 1.8242 | 1.2 | 1 | Down | 0.040675 |
| AUR62019916-RA | DUF538 family protein | 18.127 | 8.1241 | 42.5 | 6 | Down | 0.000095313 |
| AUR62036557-RA | Plant/F25P12-18 protein | 16.775 | 10.215 | 33.1 | 4 | Down | 0.000101553 |
| AUR62031146-RA | Plant/F25P12-18 protein | 16.758 | 15.277 | 26.2 | 4 | Down | 0.00003544 |
| AUR62030503-RA | UPF0098 protein CPn_0877/CP_0992/CPj0877/CpB0906-like | 18.879 | 62.629 | 44.8 | 6 | Up | 4.0973E-06 |
| AUR62030790-RA | UPF0098 protein CPn_0877/CP_0992/CPj0877/CpB0906-like | 18.865 | 19.199 | 30.8 | 5 | Up | 1.9623E-06 |
| AUR62007955-RA | Unknown protein | 20.462 | 1.2701 | 4.4 | 1 | Down | 0.00015613 |
| AUR62032243-RA | Unknown protein | 15.423 | 22.422 | 44.5 | 5 | Down | 0.000081164 |
| AUR62030980-RA | Unknown protein | 14.656 | 9.1284 | 14.2 | 2 | Down | 1.83977E-05 |
| AUR62037253-RA | Unknown protein | 20.095 | 1.6095 | 4.3 | 1 | Down | 1.5907E-06 |
| AUR62027252-RA | Unknown protein | 7.9991 | 2.3476 | 11.8 | 1 | Up | 1.3422E-06 |
| AUR62008040-RA | Unknown protein | 12.842 | 10.304 | 47.7 | 3 | Up | 0.0080976 |
| AUR62003219-RA | Unknown protein | 8.6711 | 17.606 | 49.4 | 3 | Up | 0.0175394 |
| AUR62039754-RA | Unknown protein | 13.813 | 11.009 | 24.4 | 3 | Up | 0.000062406 |
| AUR62019451-RA | Unknown protein | 18.982 | 22.928 | 20.7 | 3 | Up | 1.4059E-07 |
| AUR62001321-RA | Unknown protein | 34.238 | 13.831 | 16.5 | 5 | Up | 1.4332E-06 |
| AUR62028408-RA | Unknown protein | 14.018 | 15.47 | 17.3 | 2 | Up | 0.000001956 |
| AUR62021035-RA | Unknown protein | 7.4564 | 2.426 | 16.4 | 1 | Up | 0.00167933 |
| AUR62025657-RA | Unknown protein | 14.98 | 9.8463 | 17.8 | 2 | Up | 0.000155194 |
| AUR62022904-RA | Unknown protein | 8.6894 | 8.2482 | 34.6 | 2 | Up | 2.944E-07 |
